# Supplementary material for: Multistep diversification in spatiotemporal bacterial-phage coevolution
Source: Nat Commun. 2022 Dec 28;13:7971. doi: 10.1038/s41467-022-35351-w (PMC9797572; doi:10.1038/s41467-022-35351-w)
Supplement: Supplementary file 1 — Supplementary Information [file 41467_2022_35351_MOESM1_ESM.pdf]

Multistep diversification in spatiotemporal bacterial-phage coevolution

Supplementary Information

Einat Shaer Tamar, Roy Kishony

Supplementary Table 1 - MAGE oligos and primers used for construction of the *waaJ* and *opgG* single and double mutants.

Description	Name	Sequence
waaJ Oligo	waaJ_T877C_MODEST	5' TAAATTCAATAATTGATTTCGCATCTCGTGGAGAGTCATCTTTCCgGGGGGAATTTTCTAAAGCTATTTTATAGTATTTTACCGATGGAT 3'
waaJ MASC WT forward primer	waaJ_MASC_F_WT2	5' TCGCATCTCGTGGAGAGTCATCTTTCCA 3'
waaJ MASC Mut forward primer	waaJ_MASC_F_MUT2	5' CGCATCTCGTGGAGAGTCATCTTTCCG 3'
waaJ MASC reverse primer	waaJ_R2	5' ATGAATGTCTTACTGAAAGGGATGACATTATTTTTGCCTCG 3'
opgG Oligo	opgG_1171G->T_MODEST	5' CGTTTGTTGCACCCATGCGTTATCTGGCGCATGCAGTTTGTCTTaATCACGGCTGAAGGTGATGGTGTATTTAAAGTTCATCTCTTTACC 3'
opgG MASC WT forward primer	opgG_F_WT	5' ACTTTAAATACACCATCACCTTCAGCCGTGATG 3'
opgG MASC Mut forward primer	opgG_F_MUT	5' GAACTTTAAATACACCATCACCTTCAGCCGTGATT 3'
opgG MASC reverse primer	opgG_R	5' ACTTTCACACGCATCACCAGACGCC 3'

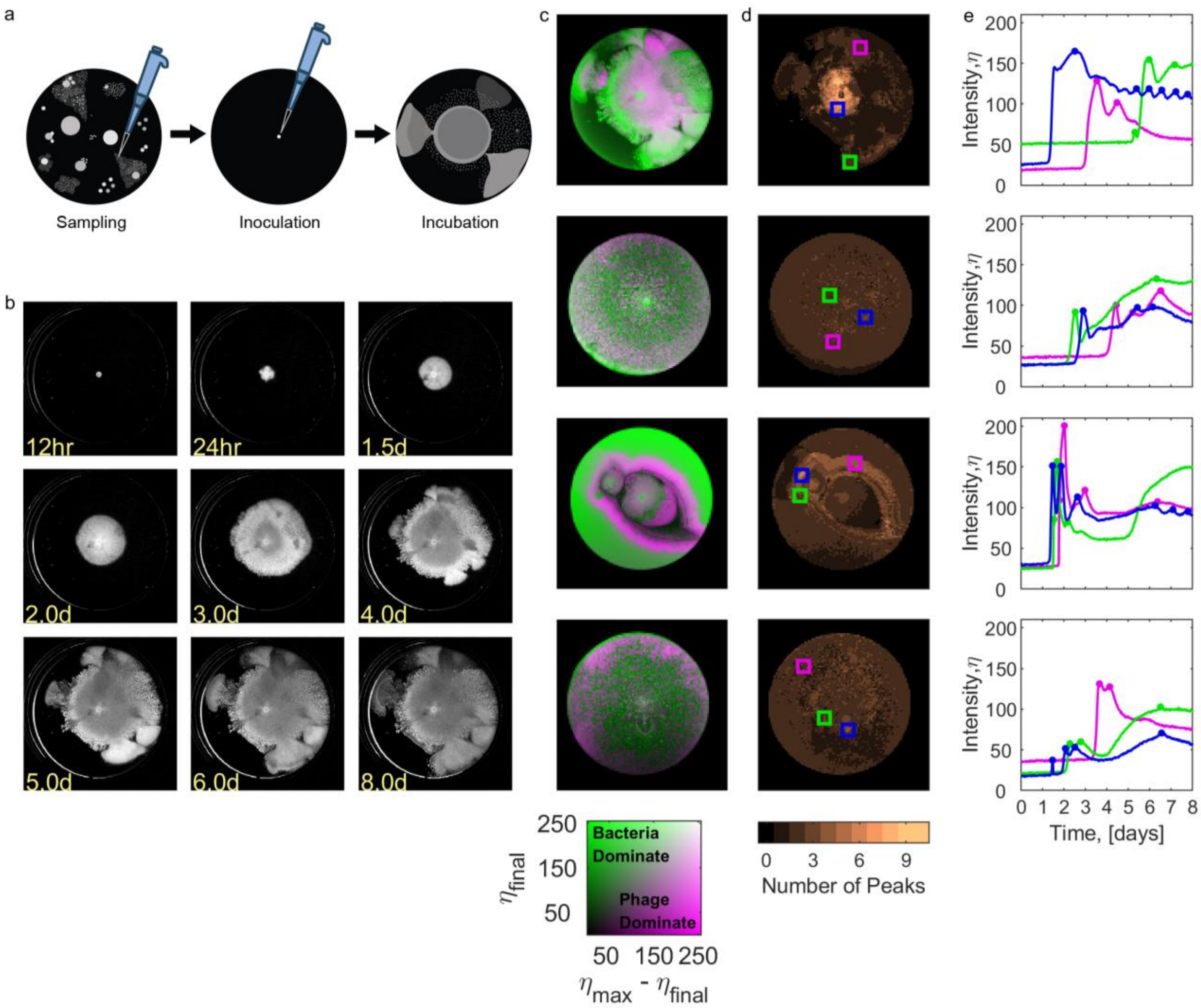

Supplementary Figure 1 - Coexistence and multiple adaptive cycles continue after re-inoculation on fresh swimming plates. a, Experimental setup of continual evolution: A sample from the endpoint of each coevolution replicate is inoculated at the center of a new swimming plate (14cm diameter, 70ml of 0.3% agar in LB) and incubated at 30C for 8 days. Note that the upper image is similar to the lower image in Fig. 1, as it represents the endpoint of the first coevolution experiment. b, Sample images of a representative replicate at different time points. c, A composite heatmap of final pixel intensity (η_{final}, green) and the difference between the maximal and final intensity (η_{max}-η_{final}, magenta), representing bacterial or phage dominance respectively on each of the four replicates at the final time point (2D legend, bottom left). d, Heatmap of the number of observed growth-infection cycles, as quantified by enumerating intensity peaks in each location. e, Intensity over time at specific locations (rectangles in d), chosen to represent areas of bacteria-dominance (green) or areas of phage-dominance (magenta) at the endpoint and highly dynamic areas with multiple intensity peaks (blue). The dots represent detected peaks. Source data are provided as a Source Data file.

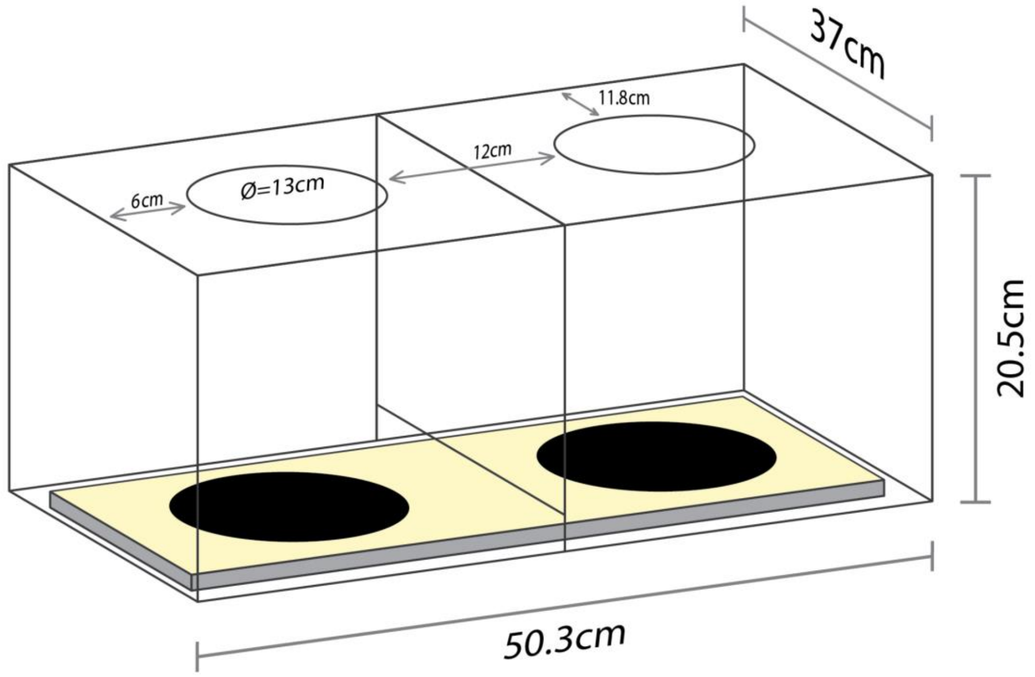

Supplementary Figure 2 - Dark field illumination setup design. Each box is designed to illuminate two swimming plates. The box is made of black acrylic glass sheets (3mm width) and is placed on top of a LED light pad (light yellow surface). A black barrier sheet in the middle prevents light from crossing between the two sides. Two circular holes were cut at the center of each half of the box upper side, and black acrylic glass discs were placed on the light pad to prevent light from traveling directly from the pad to the camera and allow dark field illumination. In each experiment, two dark field illumination boxes were placed on a dark table inside a hood with a black fabric cover. Swimming plates (14cm diameter) were placed on top of the holes and all plates were covered with a single large heated glass just after inoculation. The camera was set at the top of the hood, 143cm above the table.

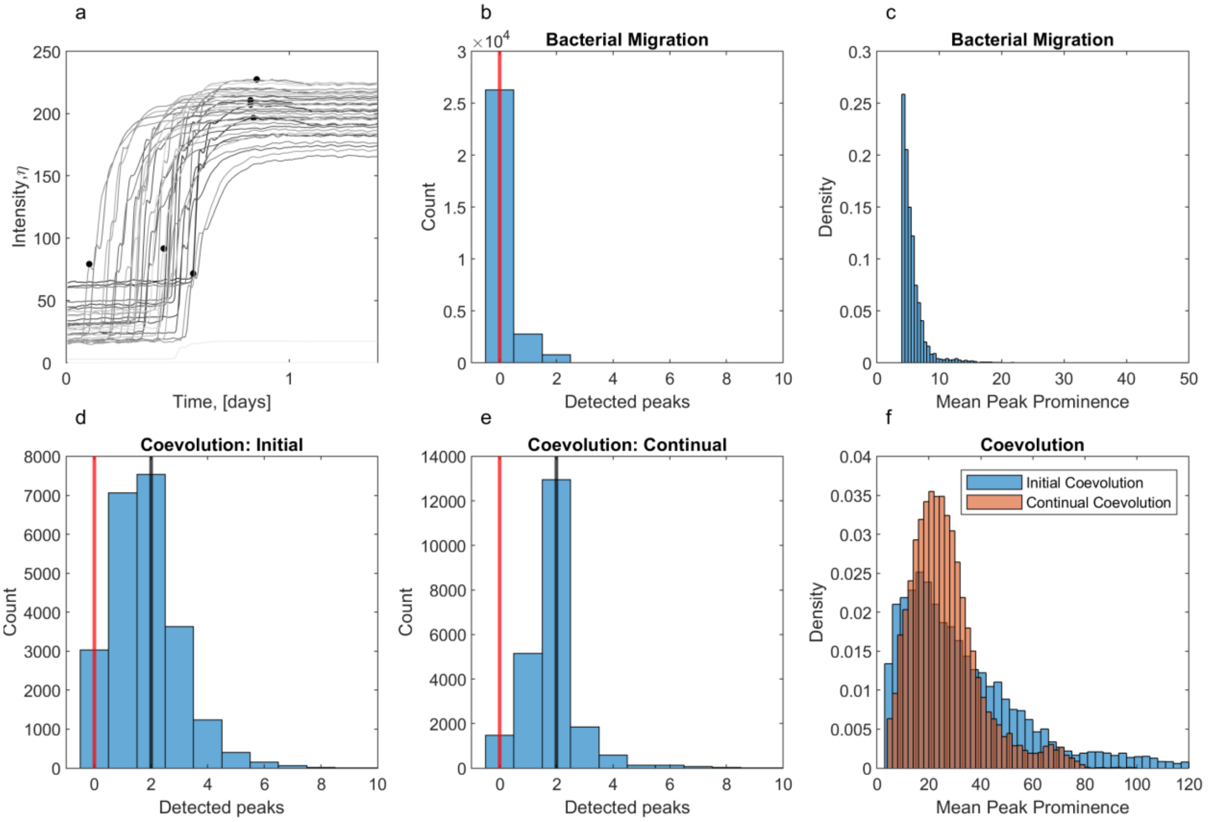

Supplementary Figure 3 - Intensity peak and peak prominence distribution in coevolution and no-phage control. a, Intensity over time during bacterial propagation on a swimming plate for 33 hours without phages, sampled at 61 random locations from replicate 1. Black dots represent detected peaks. b, Distribution of number of peaks from all four replicates during bacterial propagation. Median = 0 (red vertical line).c, Probability density function of mean peak prominence during bacterial migration: the prominence of all peaks in each point in space were averaged, and the normalized distribution of all averaged prominence values are presented. d, Distribution of number of peaks from all four replicates during the initial coevolution round, median = 2 (black vertical line, red vertical line represents median number of peaks in the no-phage control). e, Distribution of number of peaks from all four replicates during the continual coevolution round, median = 2 (black vertical line, red vertical line represents median number of peaks in the no-phage control). f, Probability density function of mean peak prominence during phage-bacteria coevolution. The prominence of all peaks in each point in space were averaged, and the normalized distribution of all averaged prominence values are presented. Light blue: initial coevolution; Orange: continual coevolution. Source data are provided as a Source Data file.

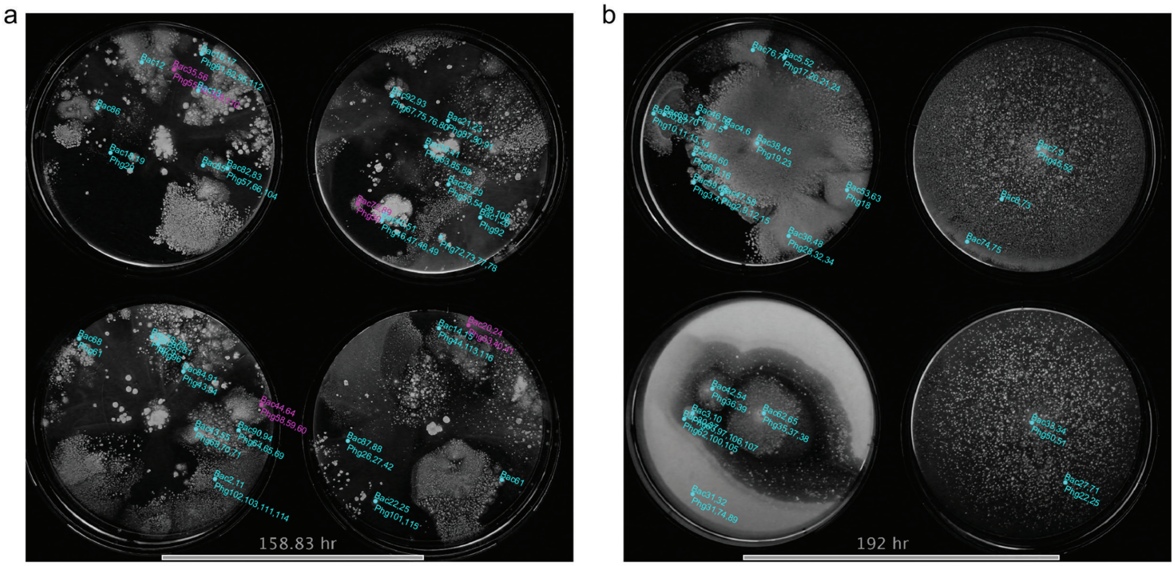

Supplementary Figure 4 - Maps of the coevolution sampled sites. Sites were samples at the end point of each coevolution round (time bar at the bottom). a, samples of the initial coevolution round. b, samples of the continual coevolution round (cyan and magenta dots). Magenta colored dots represent the samples chosen for inoculation in the continual coevolution. The names of all isolated bacteria and phages are indicated at their sampled site.

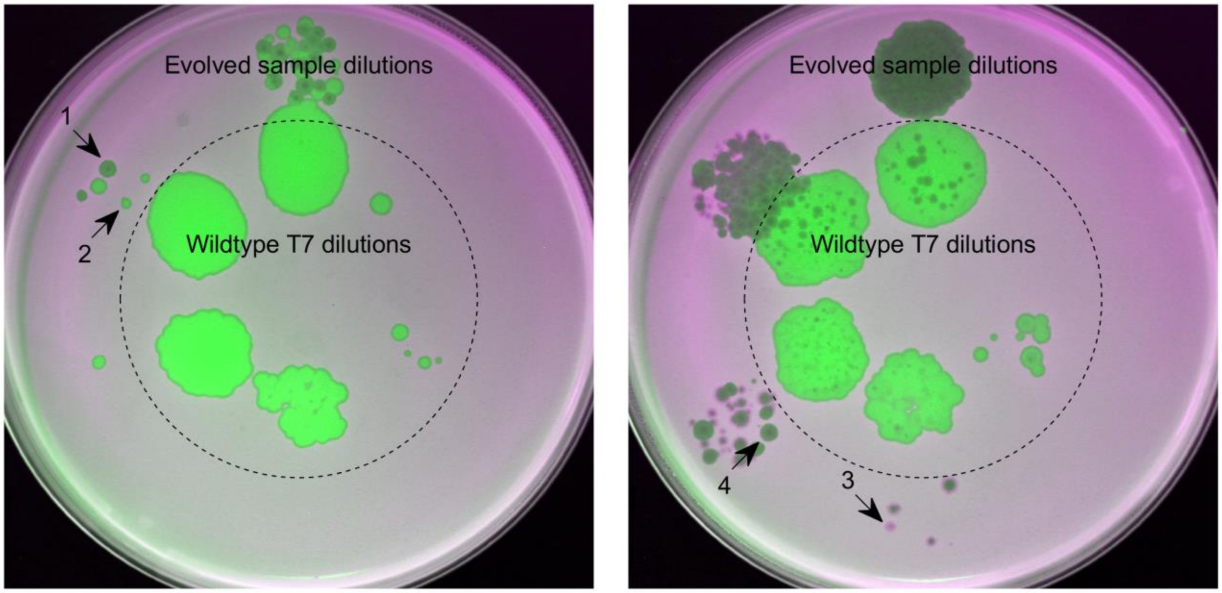

Supplementary Figure 5 - Examples of phage isolation via two-color plaque assay. The evolved bacterial isolate (YFP) and the bacterial ancestor strain MG1655 (mCherry) are mixed in soft agar (0.7%) and overlaid on an agar plate. Plates were divided into 6 equal segments and 10µl spots of six 10-serial dilutions of the evolved phage from the sample of the bacterial isolate are spotted in the outer circle, and similar dilutions of the wildtype T7 phage are spotted in the inner circle. After incubation, plates are imaged and plaques of three colors can be observed; Green: Phages that can only infect the wildtype strain (2). Black: phages that can infect both wildtype and evolved strains (1,4), Magenta (rare): phages that infect the evolved strain but not the wildtype (3, host-switch).

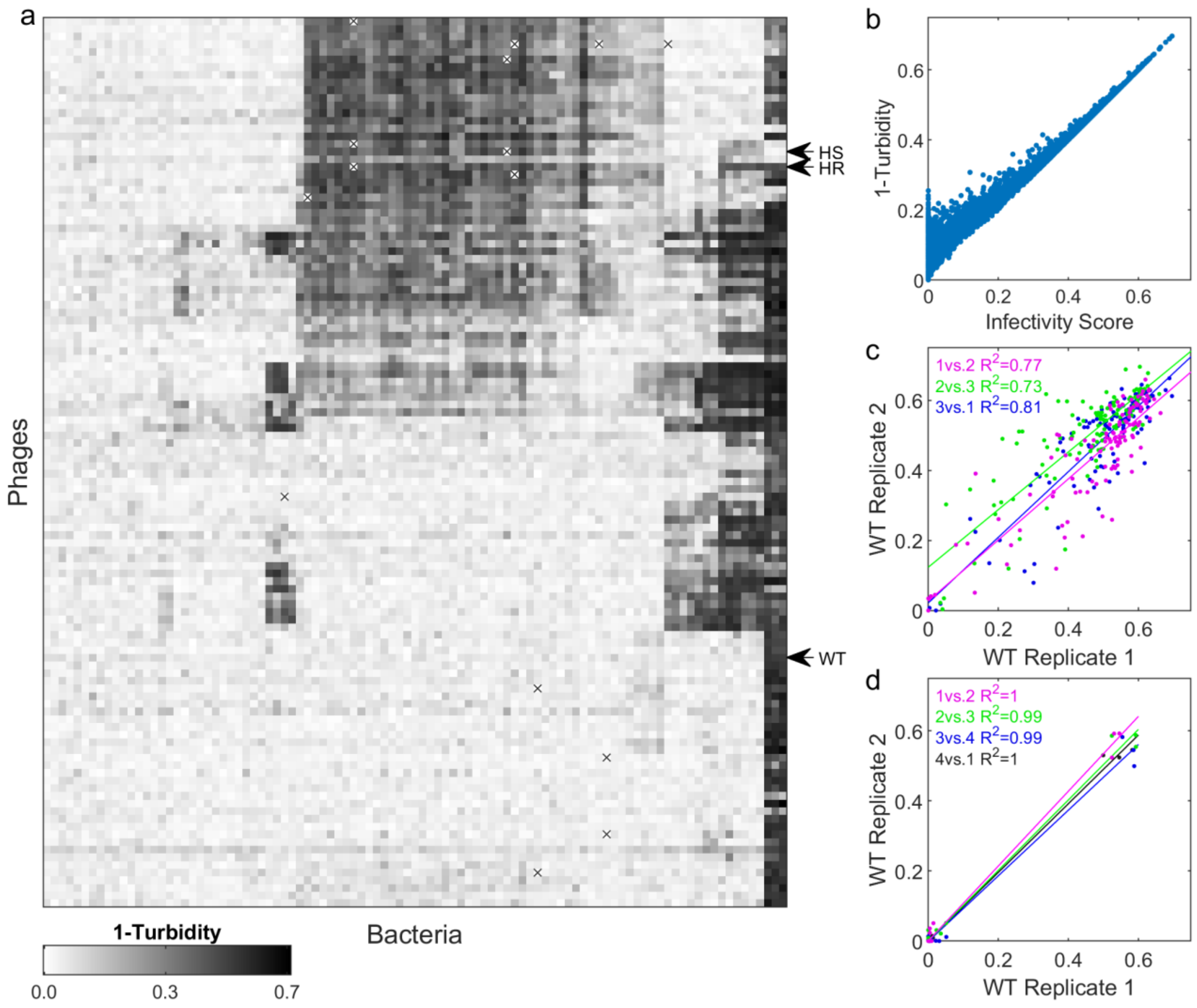

Supplementary Figure 6 - Cross infection measurement via turbidity score. a, Infection results of the cross-infection assay of all phage isolates (rows) on all bacterial isolates (columns). Points in the matrix indicate 1 - Turbidity measurements (Methods). Missing data points are marked with x. Isolates are ordered by the same isolate order as in Figure 2. WT (wildtype), HS (host switch) and HR (host range) refer to the same phage isolates marked in Figure 2a. b, All Infectivity scores plotted against all 1-Turbidity scores. c-d, correlation between the infectivity scores calculated for different replicates of the bacterial wildtype (c, 3 replicates) and the phage wildtype (d, 4 replicates), each color represents a comparison between two wildtype replicates, the solid lines show a linear fit. Source data are provided as a Source Data file.

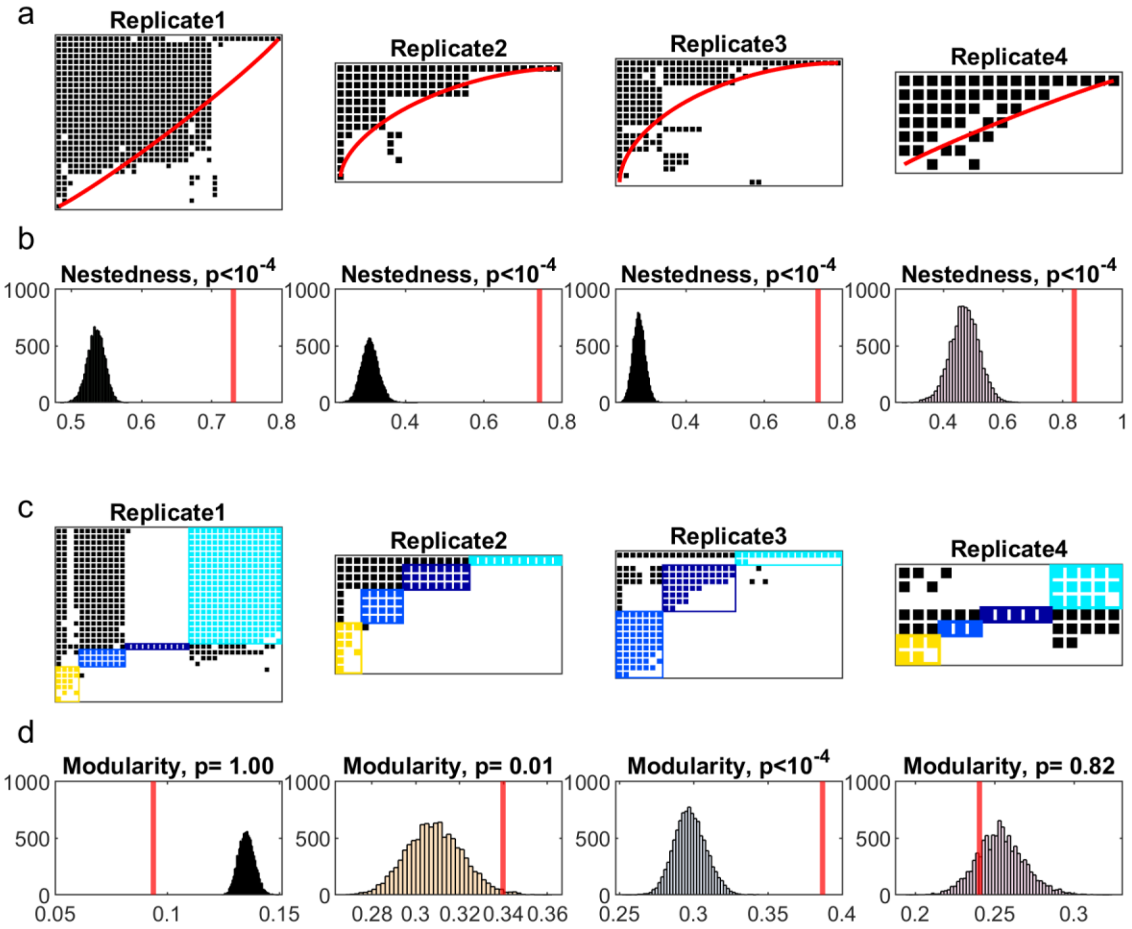

Supplementary Figure 7 - Patterns of nestedness and modularity in the interaction network of coevolved bacteria and phage. BiMat[1] analysis of the cross-infection network among isolates of each coevolution replicate (including isolates from both initial and continual coevolution rounds; Methods, BiMat analysis). a, Cross-infection matrices are sorted according to the NODF algorithm to show nestedness. b, nestedness value of the corresponding replicate matrix (red line) and the distribution of nestedness values among 10,000 randomly reordered matrices constructed with the EQUIPROBABLE null model. Statistical significance was calculated as the chance to get equal or larger nestedness values. c, Cross-infection matrices are clustered according to the adaptive BRIM algorithm to show modularity. d, modularity of the corresponding replicate matrix (red line) and the distribution of modularity values among 10,000 randomly reordered matrices constructed with the EQUIPROBABLE null model. Statistical significance was calculated as the chance to get equal or larger modularity values. Source data are provided as a Source Data file.

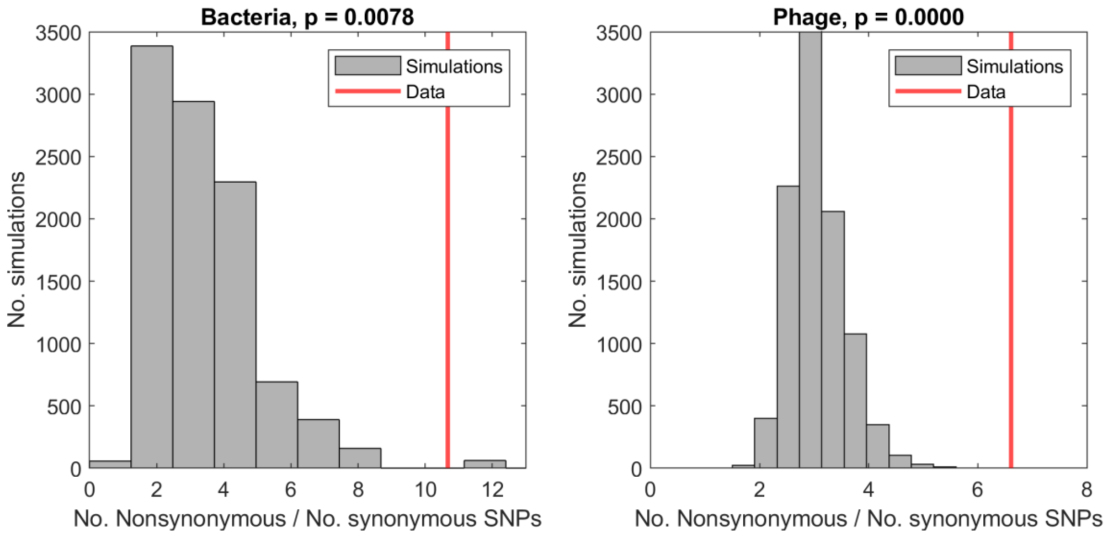

Supplementary Figure 8 - Nonsynonymous to synonymous mutation ratios demonstrate adaptive evolution. The ratio of nonsynonymous to synonymous SNPs (dN/dS) was calculated per organism (red vertical lines, left: bacteria, right: phages), and compared to the results of 10,000 simulations with randomly assigned SNP positions. The statistical significance estimator p represents the relative number of simulations with dN/dS higher than the experimental value. Source data are provided as a Source Data file.

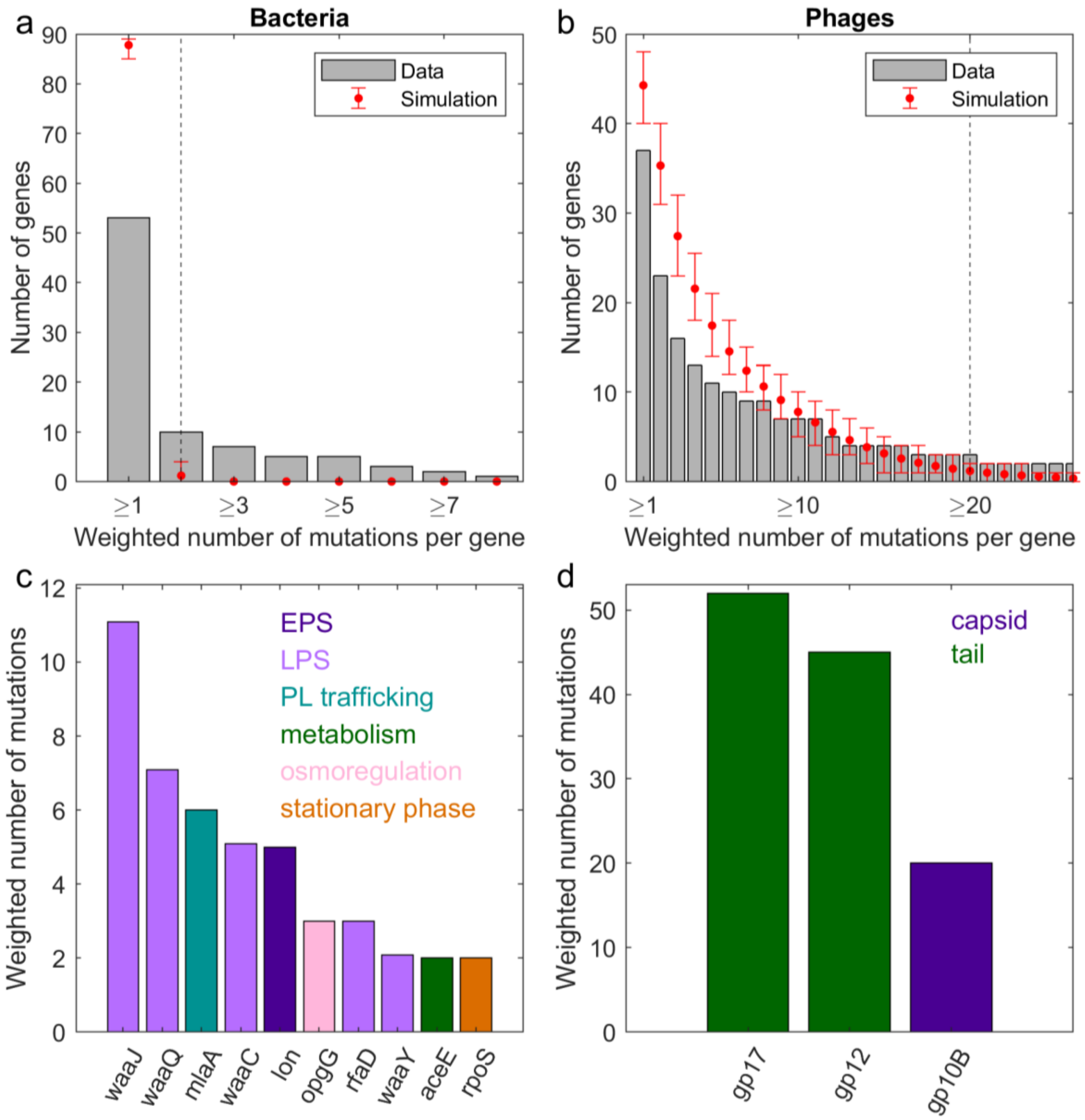

Supplementary Figure 9 - Genes mutated more than expected by chance.
a-b, The cumulative distribution of weighted number of mutations per gene (gray bars) is calculated by identifying all bacterial (a) or phage (b) genes affected by each mutation and normalizing for mutation length. The expected number of mutations per gene (red dots) is calculated by randomly distributing the original mutation set across the bacterial (a, n=500 simulation runs) or phage (b, n=5000 simulation runs) genome (Methods, Multiply-mutated genes). Error bars represent 95% confidence intervals around the mean. The multiply-mutated threshold, represented by the dashed vertical line, marks the first measured weighted number of mutations that fell below 95% of the simulations. c-d, Genes with a weighted number of mutations higher than the multiply-mutated threshold are plotted for bacteria (c) and phages (d), along with their weighted number of mutation values. Bar colors represent the biological pathway of each gene and match the color map in Figure 3. Source data are provided as a Source Data file.

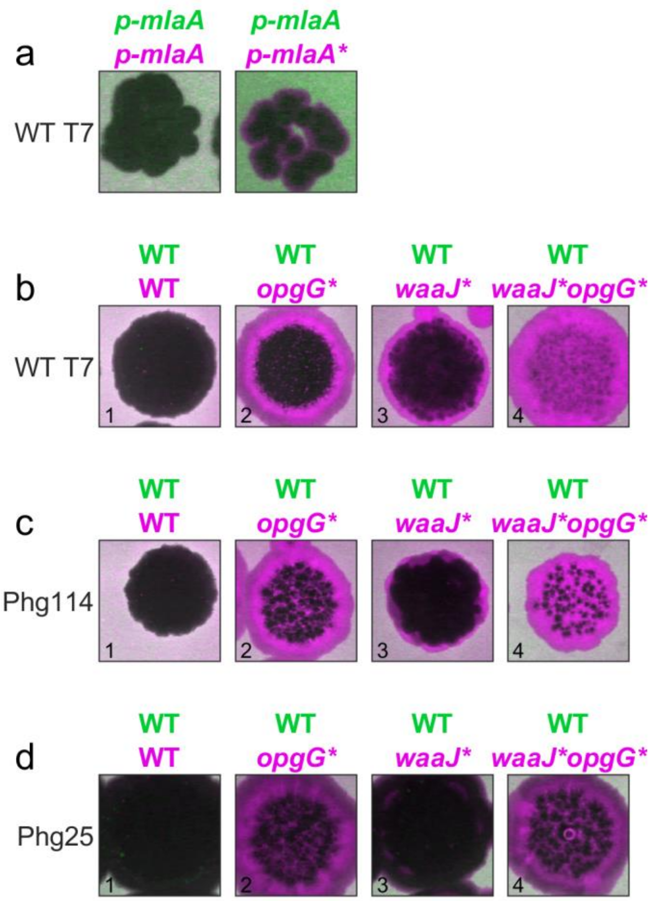

Supplementary Figure 10 - Reconstructing mutations in *opgG* or *mlaA* - Strain Dye swap of Figure 4. a, a two-color plaque assay with the wildtype T7 phage (WT T7) spotted on a mixture of YFP and mCherry tagged wildtype bacterial strains carrying a plasmid expressing either *mlaA* wildtype (p-mlaA) or *mlaA* mutant (p-mlaA*) genes[2]. Left: a control with both strains expressing the wildtype *mlaA*; Right: a strain expressing mlaA* and tagged mCherry (magenta) shows a small selective advantage over a strain expressing the wildtype *mlaA* (tagged YFP, green), manifesting as a magenta halo at the border of the plaque zone (Methods). b, An *opgG* mutant has a mild T7 resistance advantage over the wildtype (compare panel b2 versus wild-type control, panel b1) and strong synergy with the *waaJ* mutation (compare double mutant in panel b4 versus single mutants in panels b2, b3). c,d, When competed in the presence of two chosen evolved T7 phages (Phg114, c; Phg25, d; see Supplementary Table 4,5 for genotypes and phenotypes, respectively), the *opgG* mutation provides strong resistance both alone (c2, d2) and when combined with the *waaJ* mutation (c4, d4), indicating phage-specific advantage, **Please note that the p-mlaA with p-mlaA image (a, left) and WT with WT images (panel 1 in b-d) are the same images as in the main Fig. 4 as they function as wildtype controls for both dye-swap experiments, which were done at the same day.**

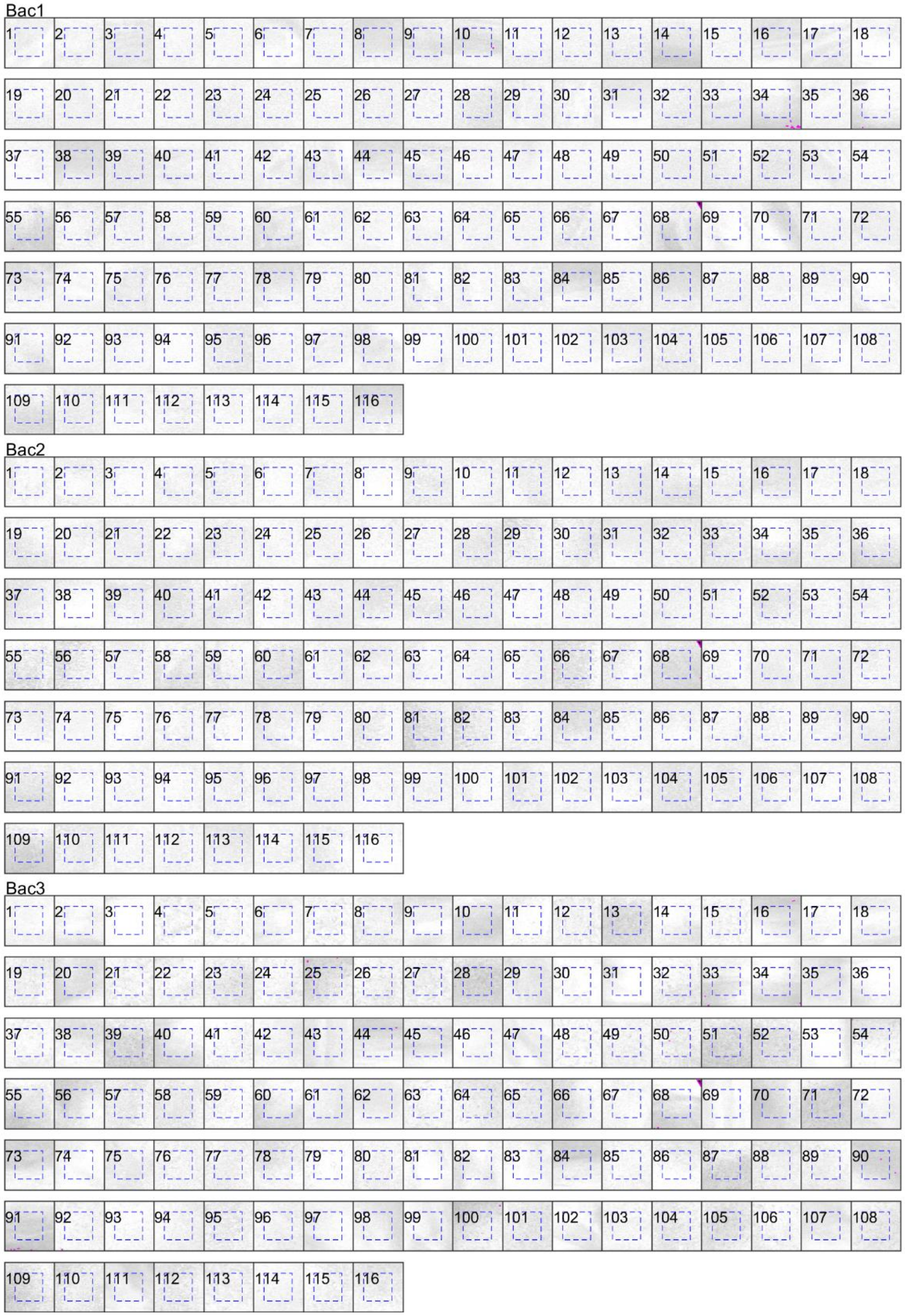

Supplementary Figure 11 - Bacteria-phage cross-infection interactions- raw data. Pink boundary lines represent detected plaque area. Infectivity is calculated within the dashed blue rectangle. **1/33**

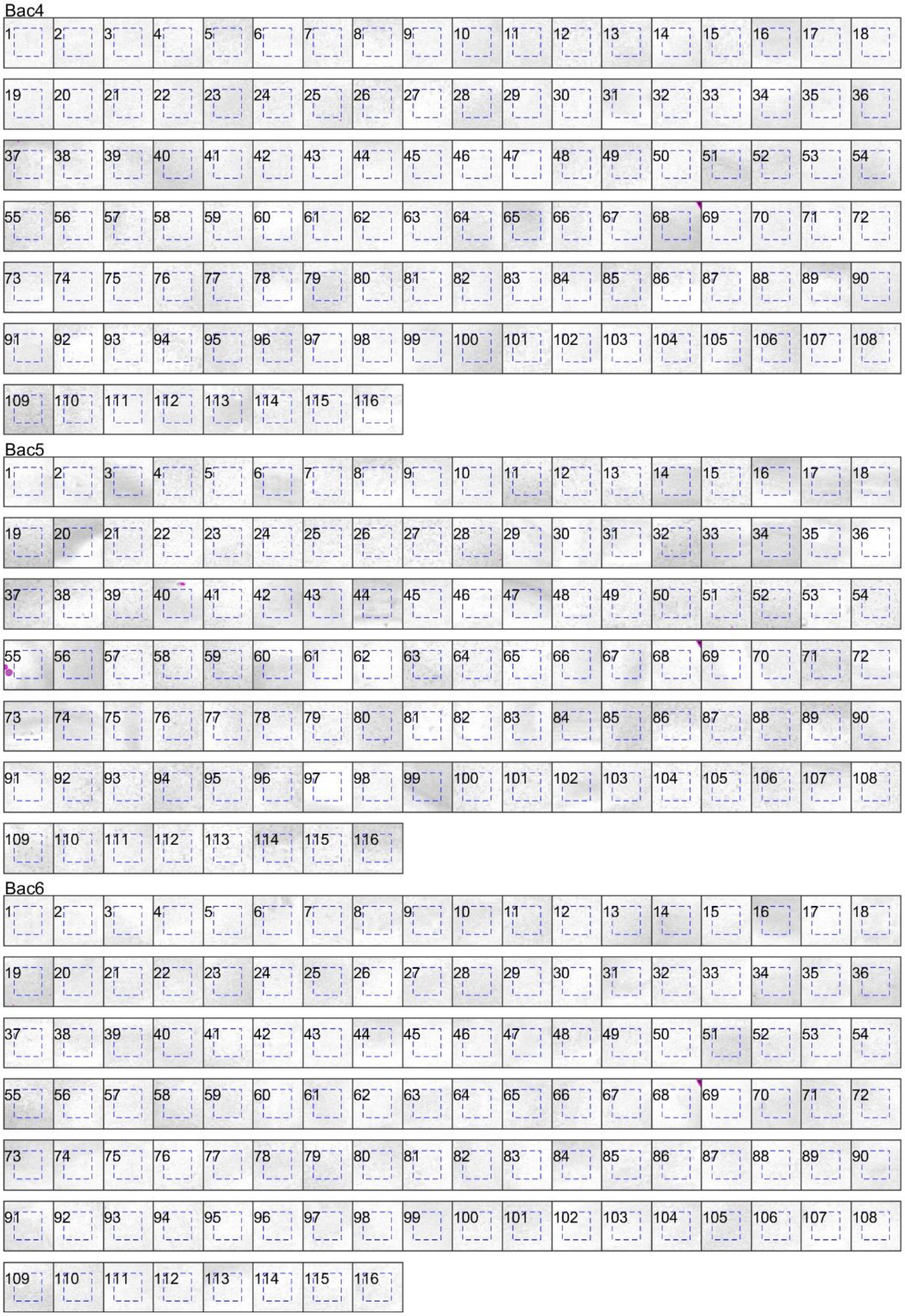

Supplementary Figure 11 - Bacteria-phage cross-infection interactions- raw data. **2/33**

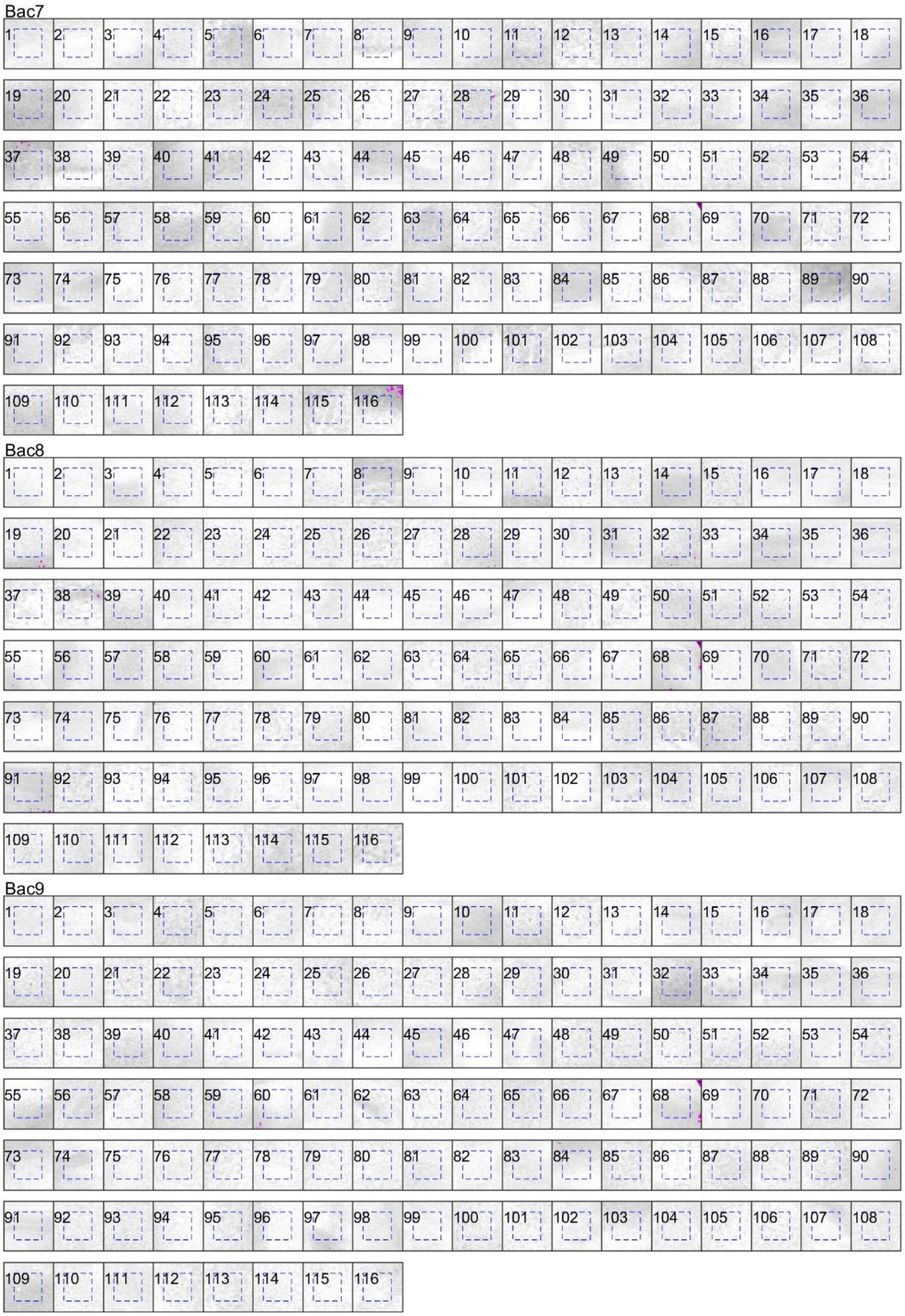

Supplementary Figure 11 - Bacteria-phage cross-infection interactions- raw data. **3/33**

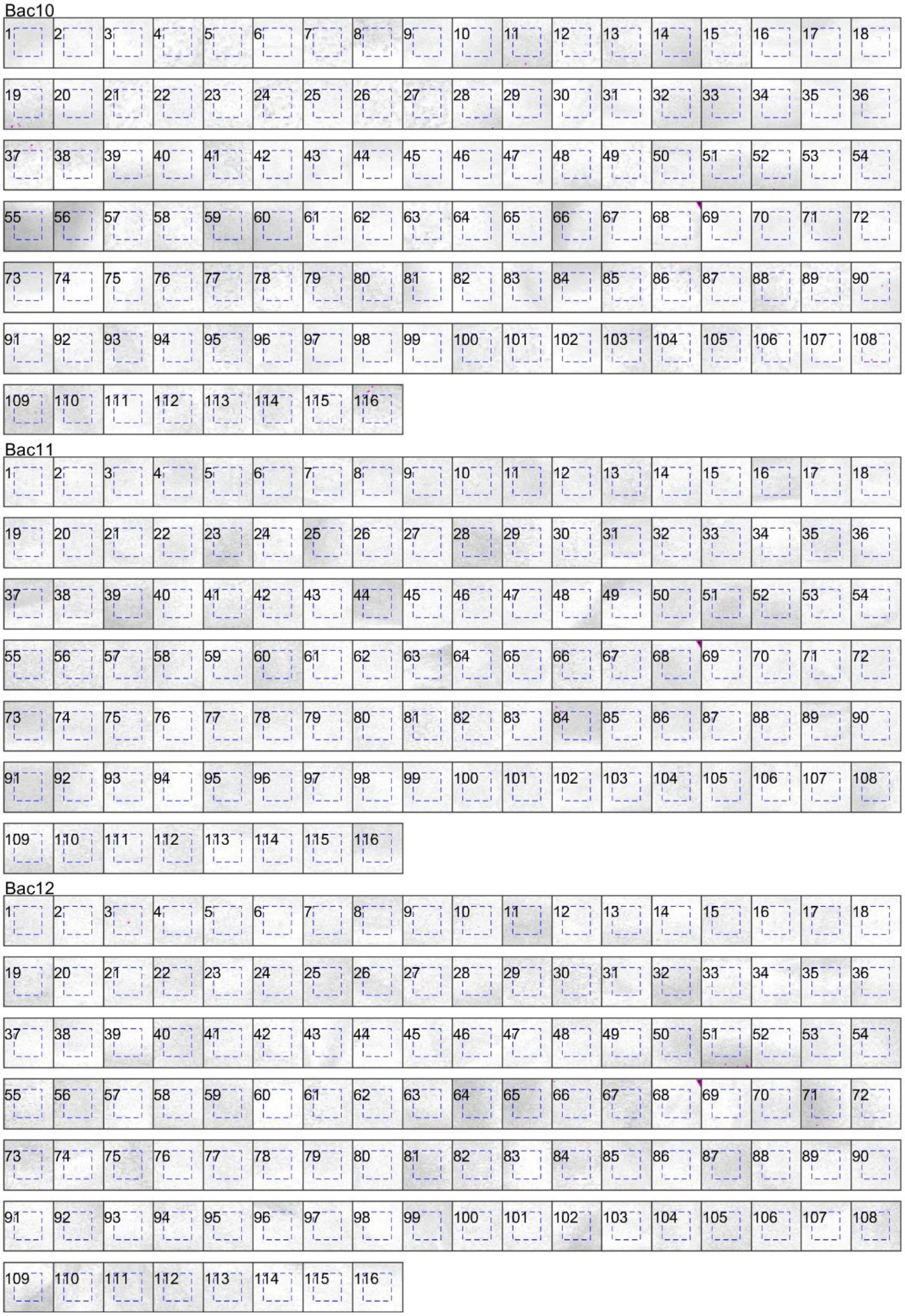

Supplementary Figure 11 - Bacteria-phage cross-infection interactions- raw data. **4/33**

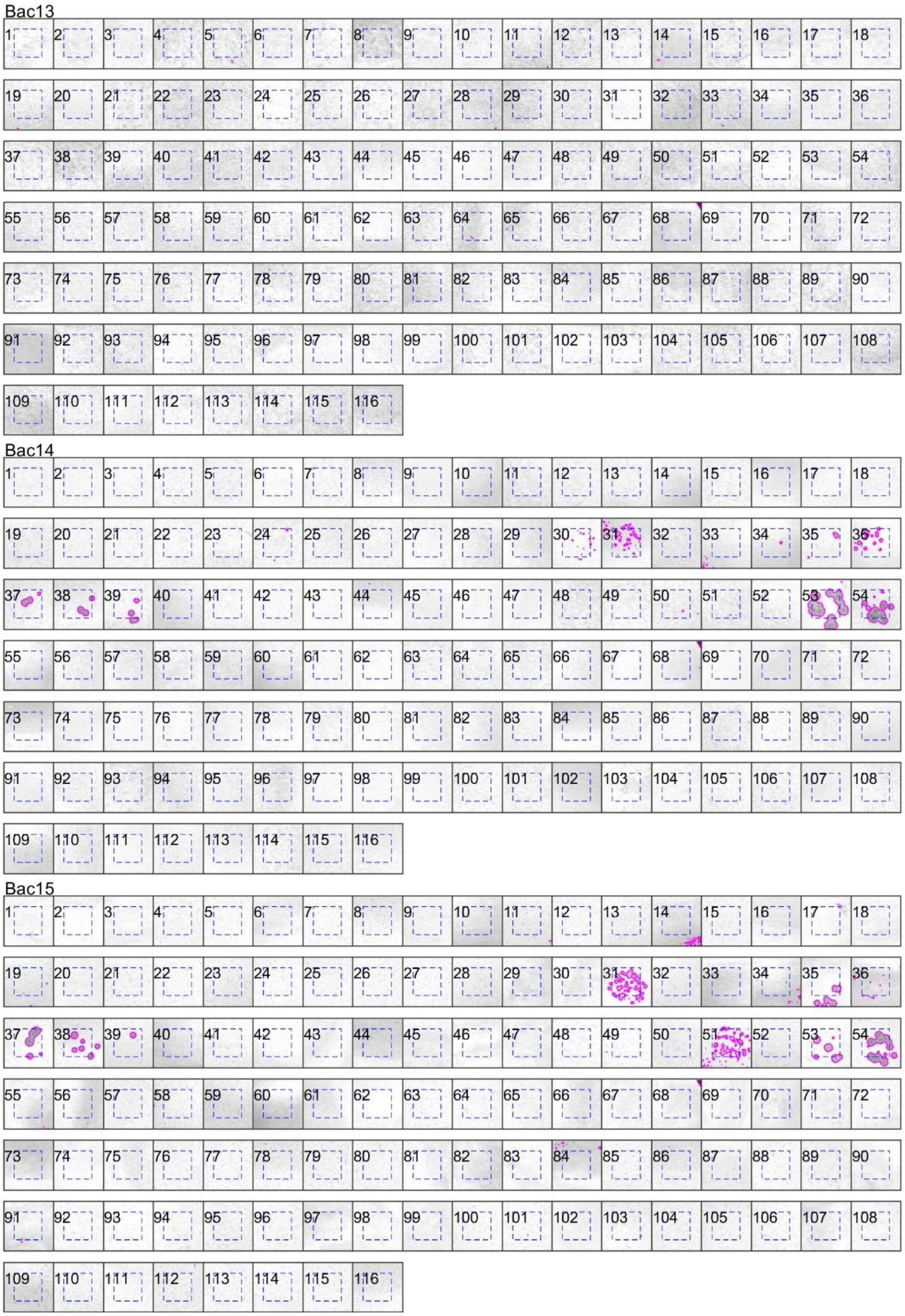

Supplementary Figure 11 - Bacteria-phage cross-infection interactions- raw data. **5/33**

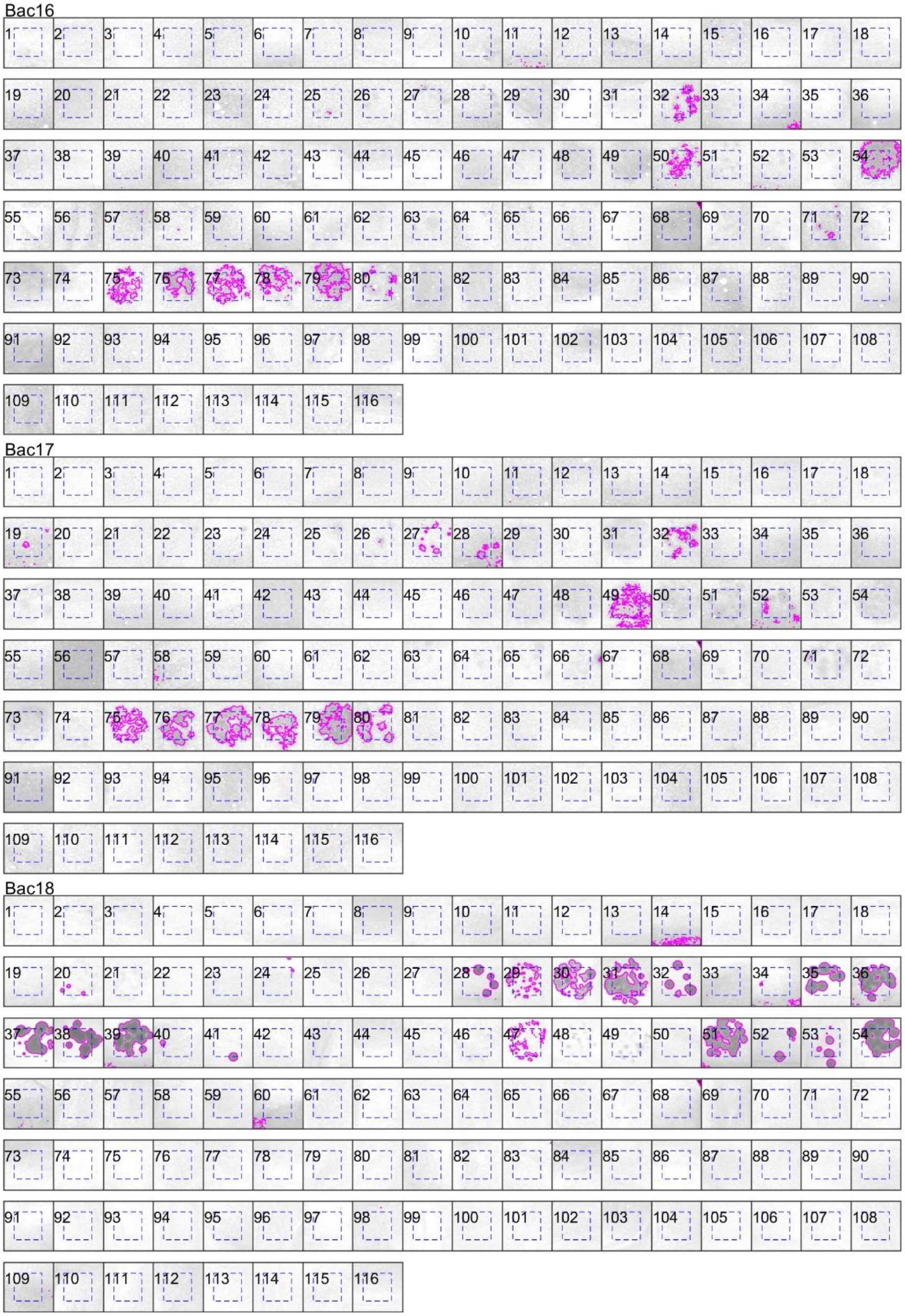

Supplementary Figure 11 - Bacteria-phage cross-infection interactions- raw data. **6/33**

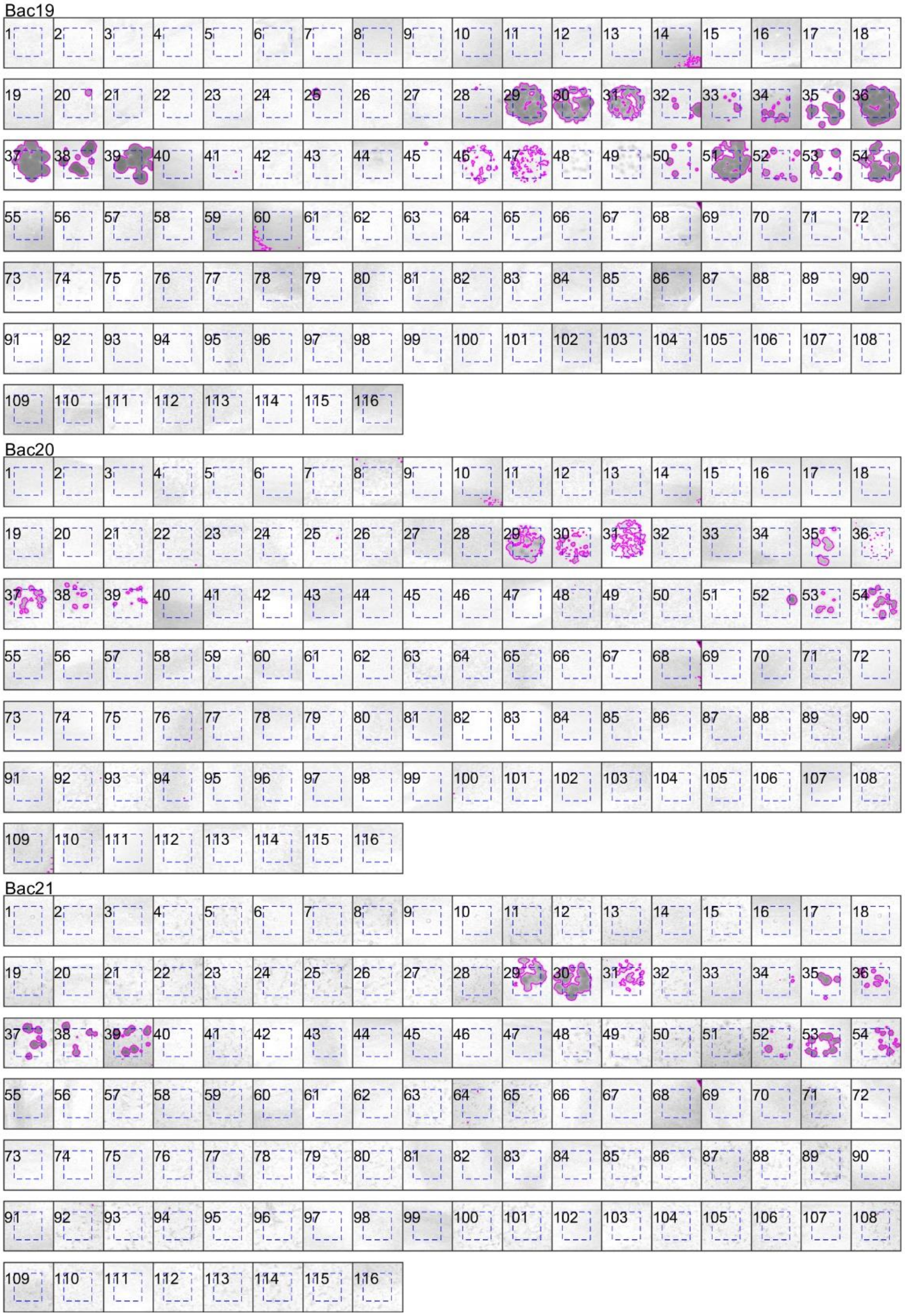

Supplementary Figure 11 - Bacteria-phage cross-infection interactions- raw data. **7/33**

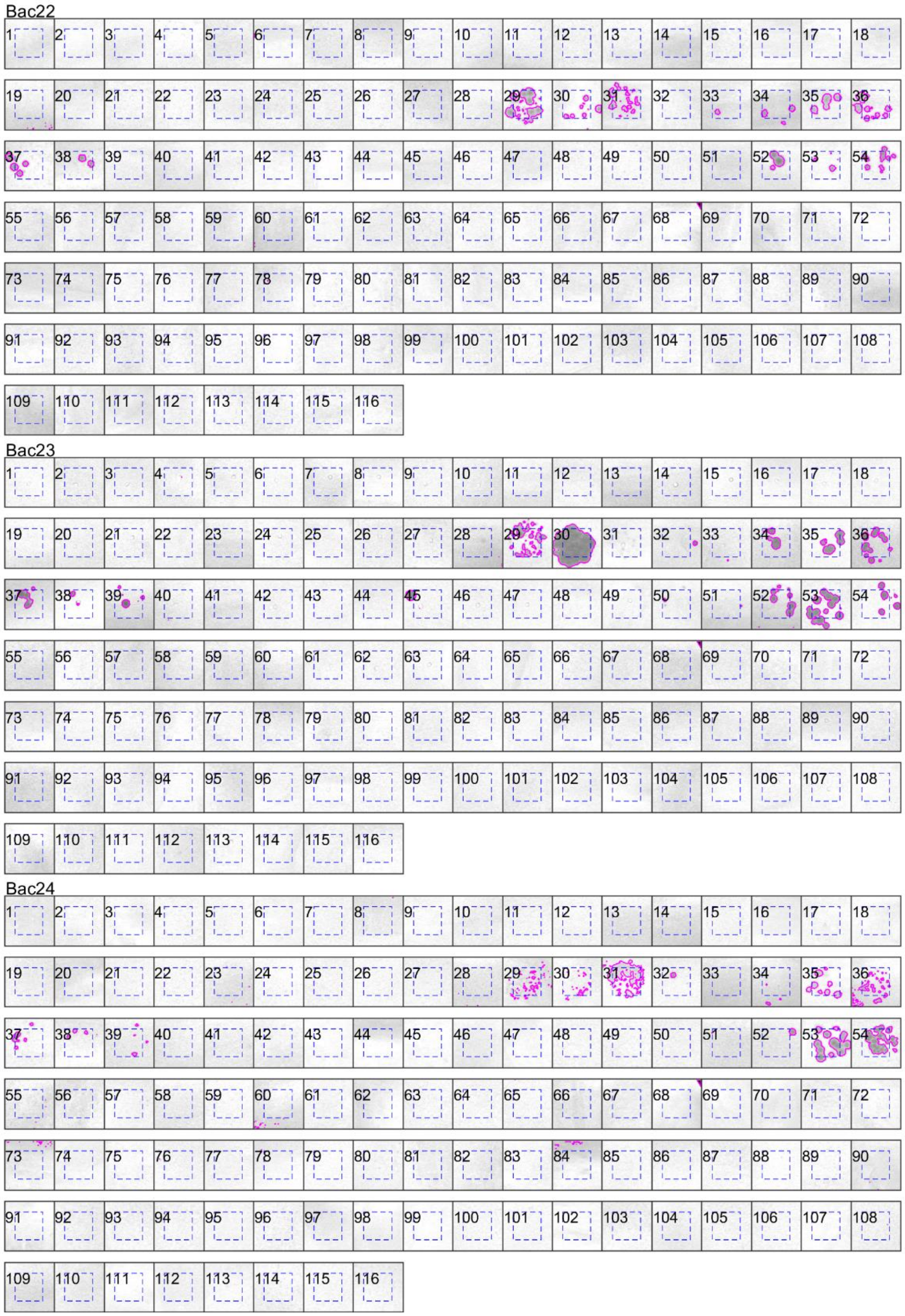

Supplementary Figure 11 - Bacteria-phage cross-infection interactions- raw data. **8/33**

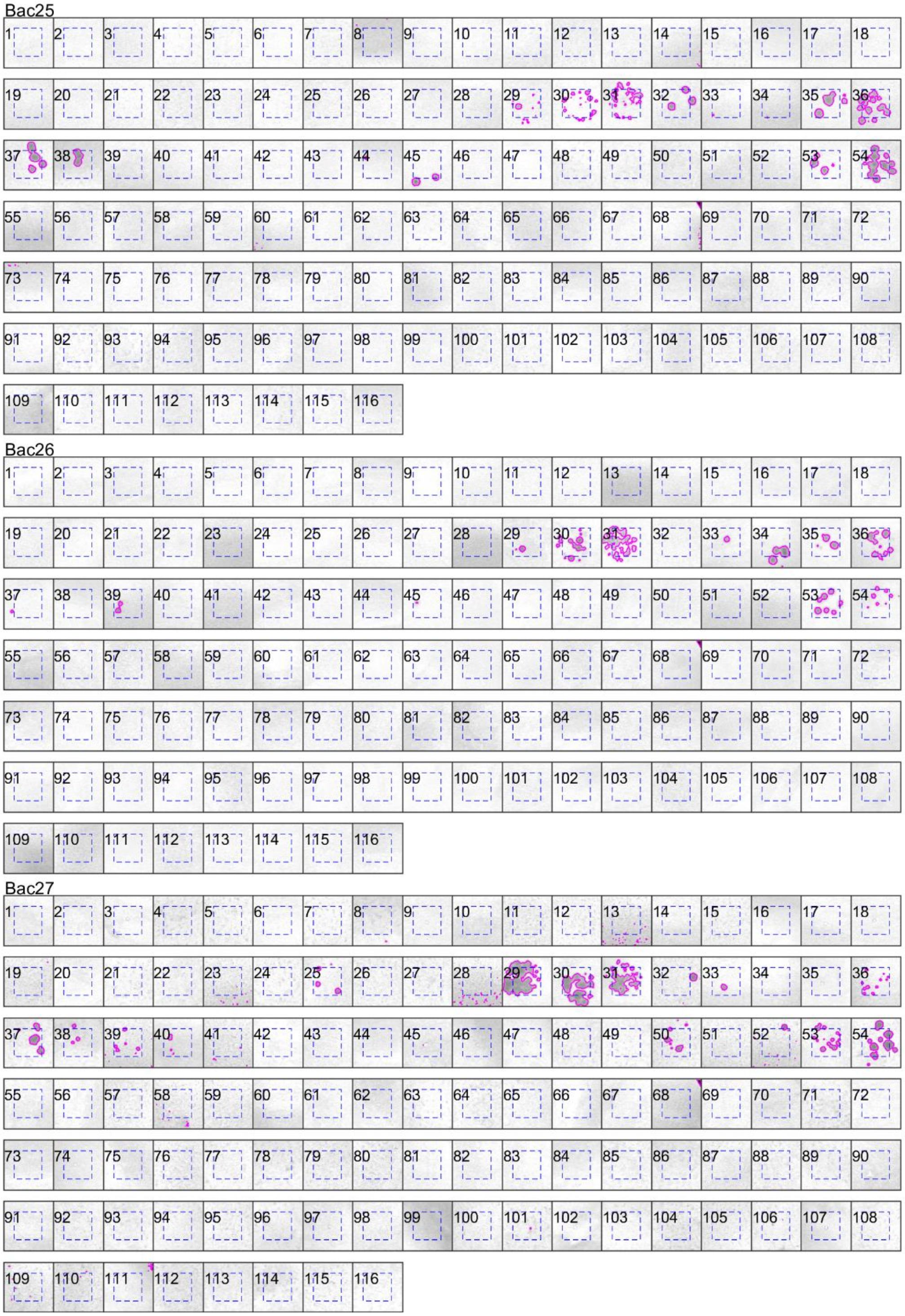

Supplementary Figure 11 - Bacteria-phage cross-infection interactions- raw data. **9/33**

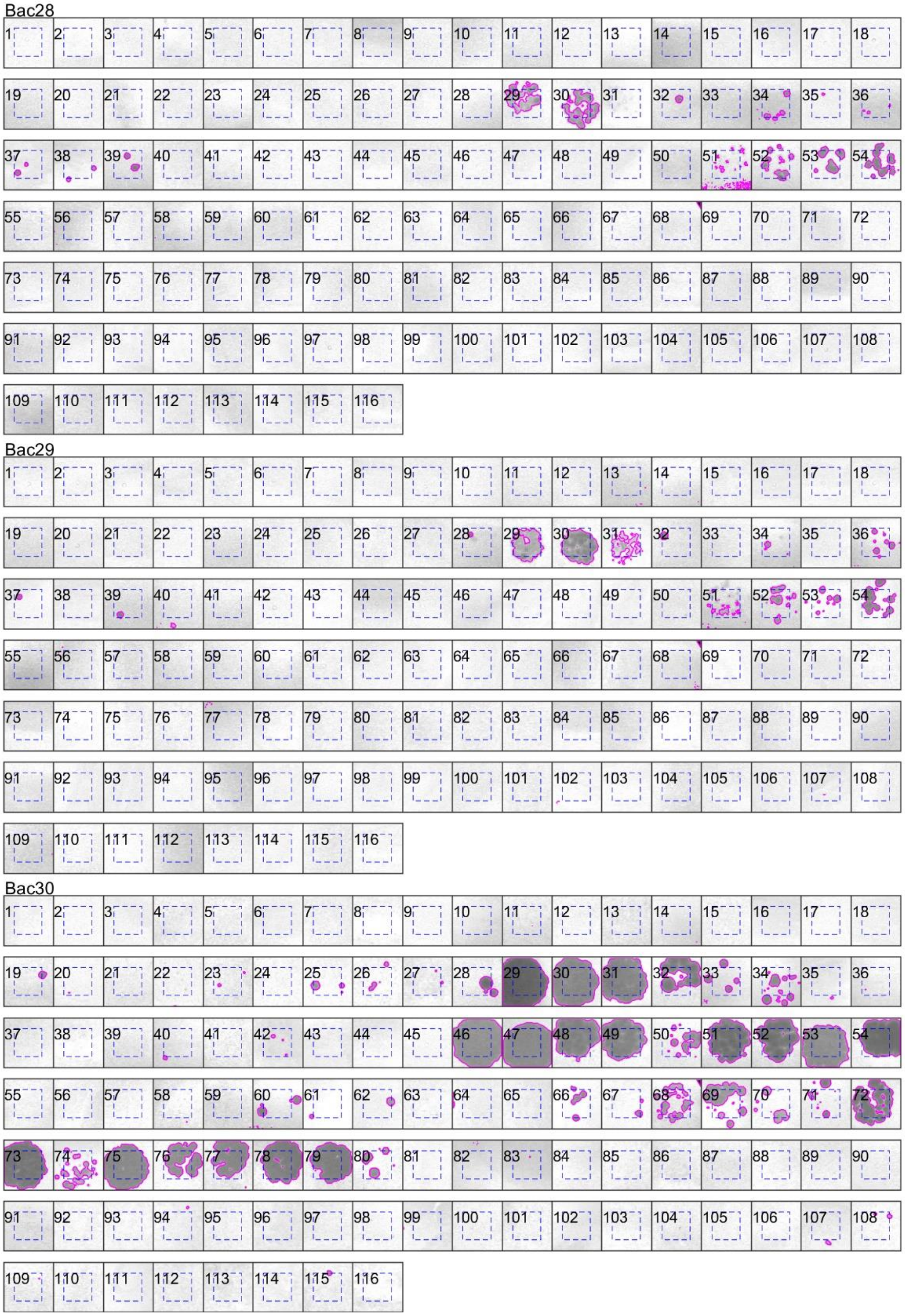

Supplementary Figure 11 - Bacteria-phage cross-infection interactions- raw data. **10/33**

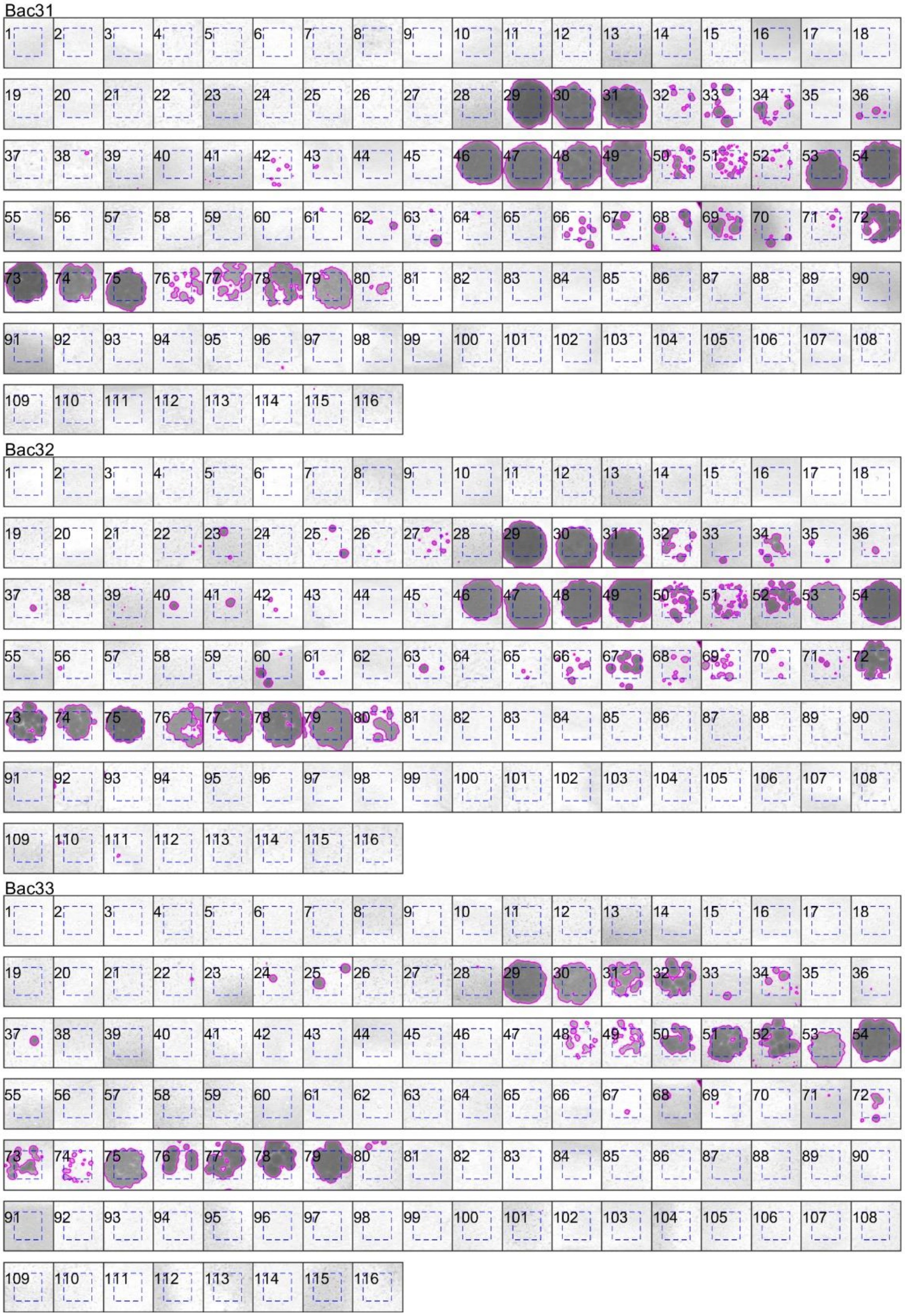

Supplementary Figure 11 - Bacteria-phage cross-infection interactions- raw data. **11/33**

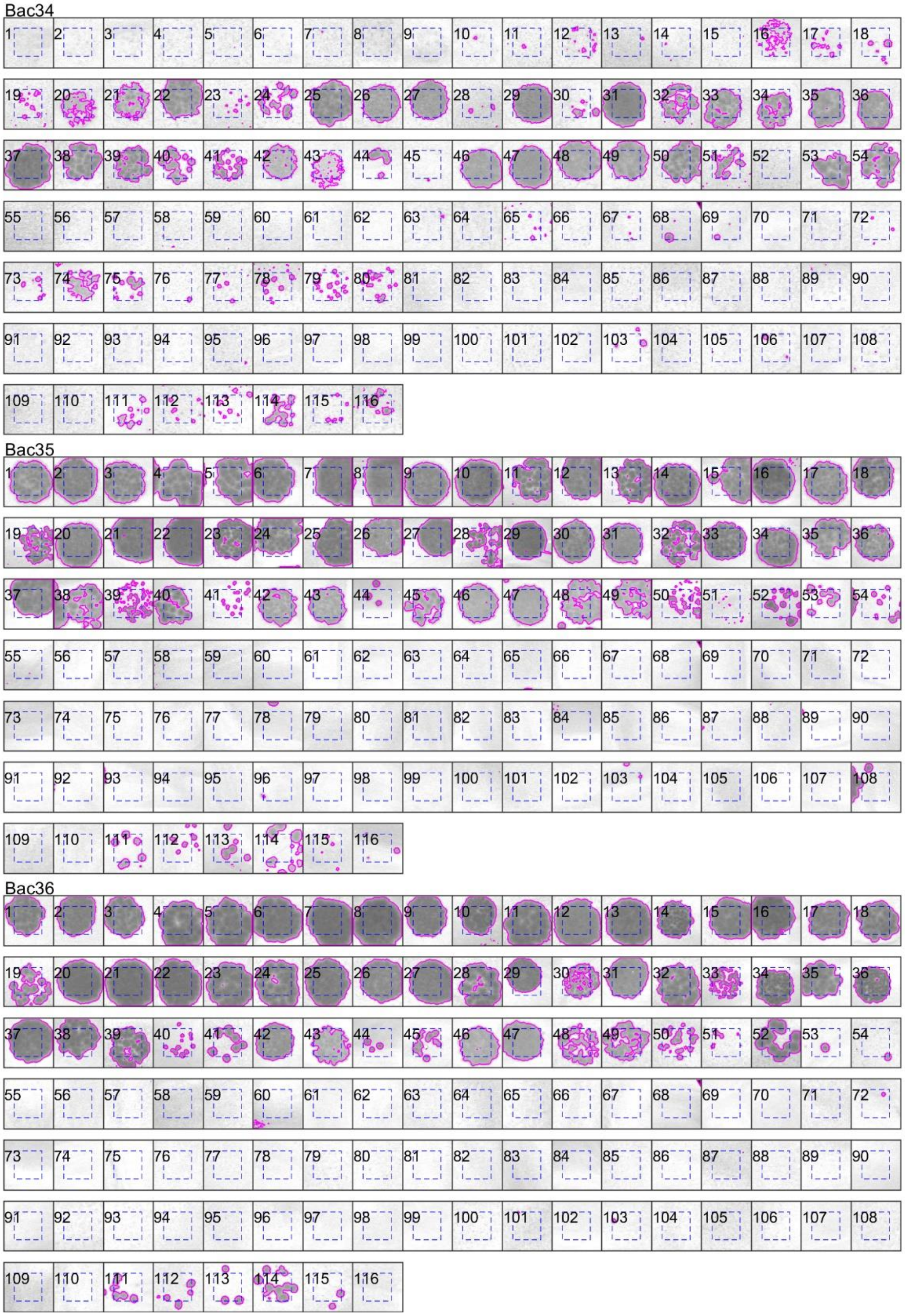

Supplementary Figure 11 - Bacteria-phage cross-infection interactions- raw data. **12/33**

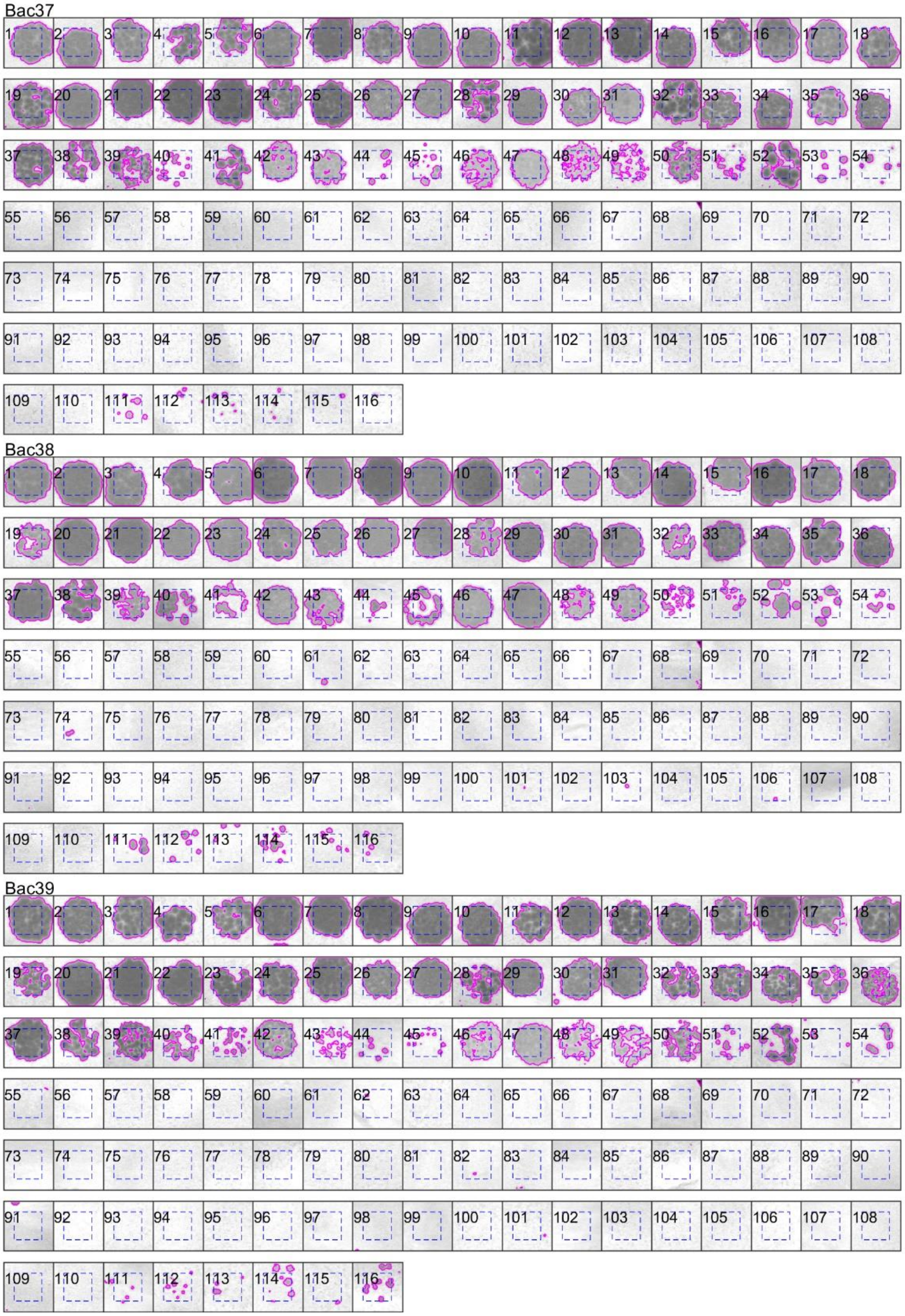

Supplementary Figure 11 - Bacteria-phage cross-infection interactions- raw data. **13/33**

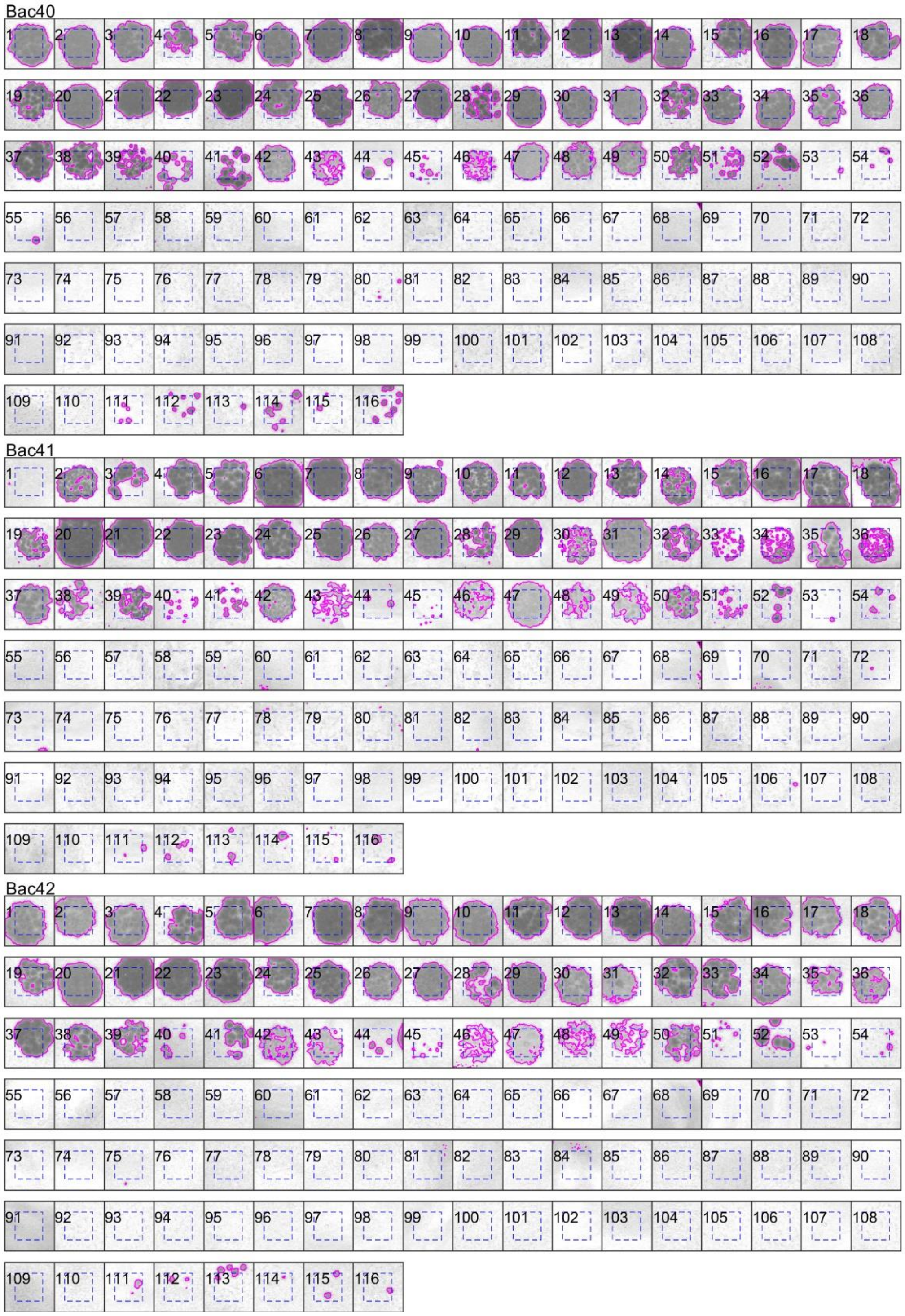

Supplementary Figure 11 - Bacteria-phage cross-infection interactions- raw data. **14/33**

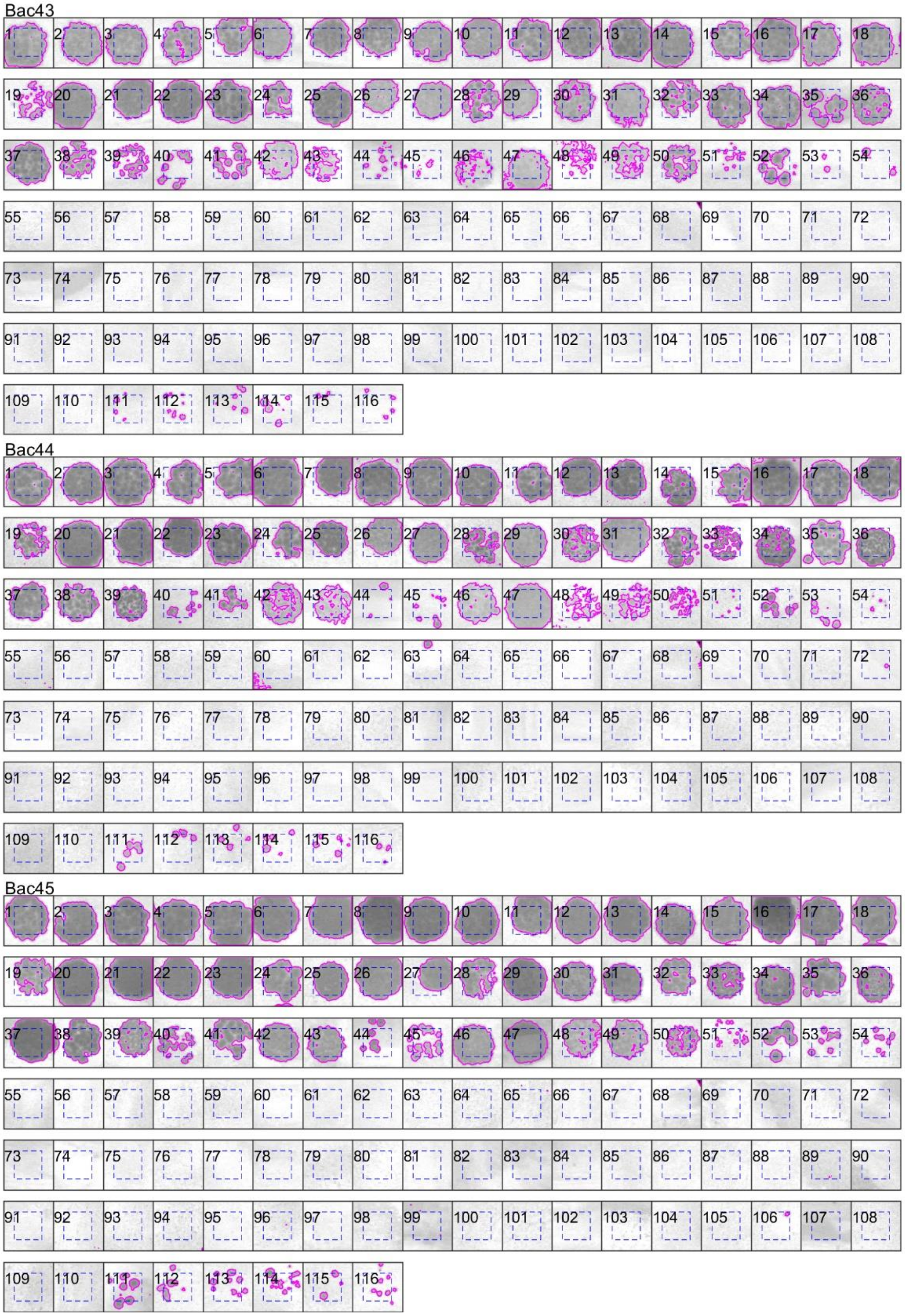

Supplementary Figure 11 - Bacteria-phage cross-infection interactions- raw data. **15/33**

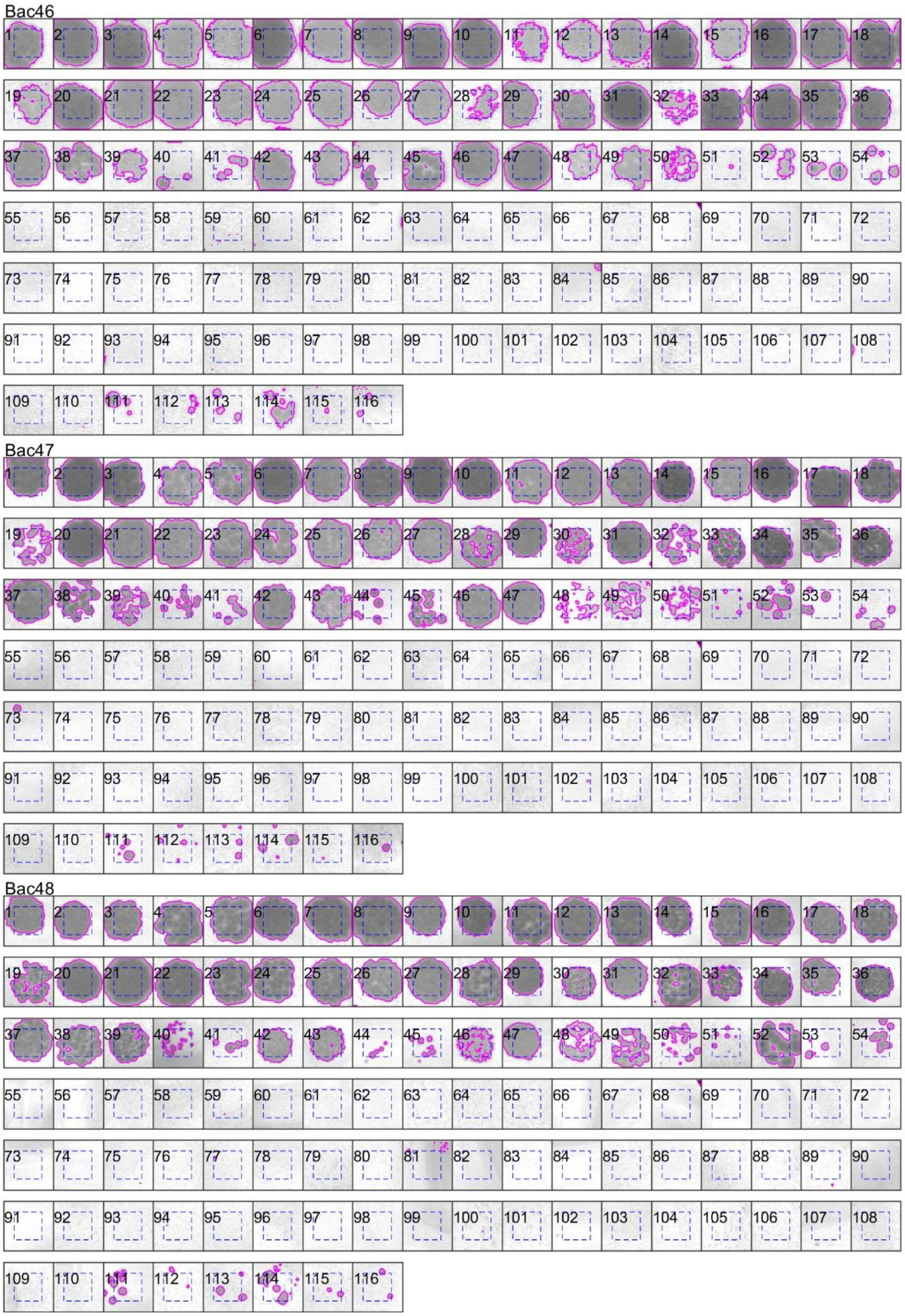

Supplementary Figure 11 - Bacteria-phage cross-infection interactions- raw data. **16/33**

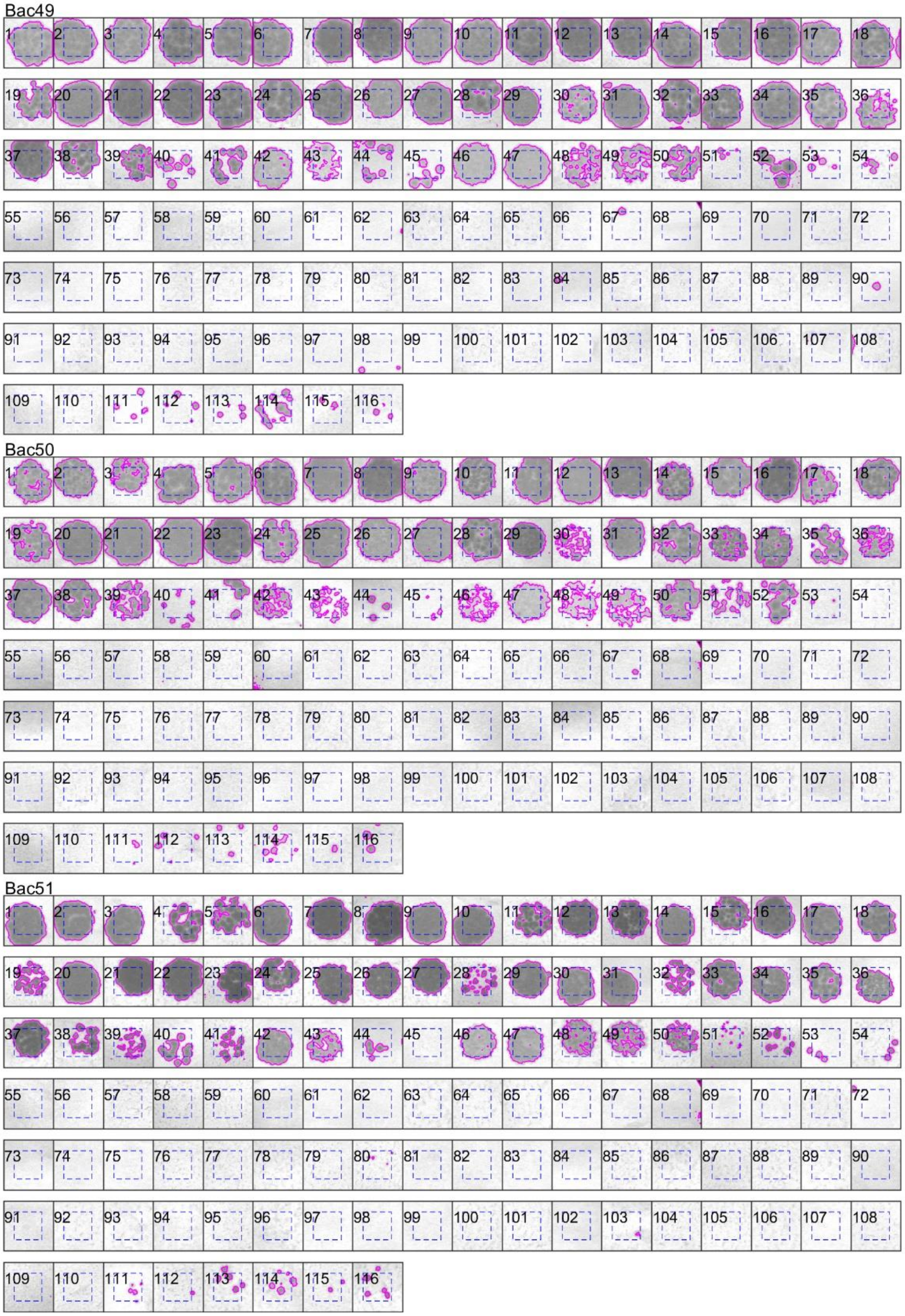

Supplementary Figure 11 - Bacteria-phage cross-infection interactions- raw data. **17/33**

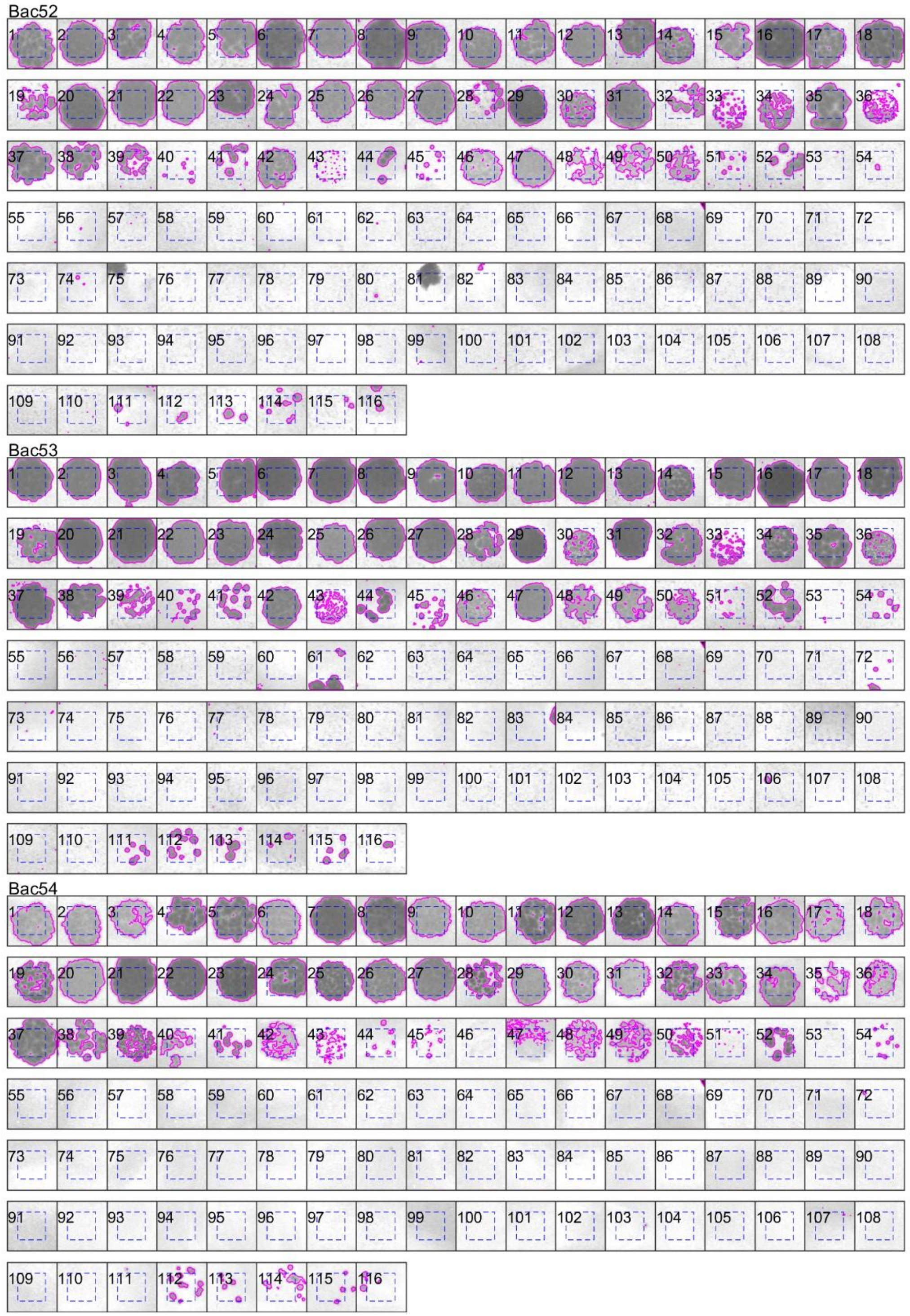

Supplementary Figure 11 - Bacteria-phage cross-infection interactions- raw data. **18/33**

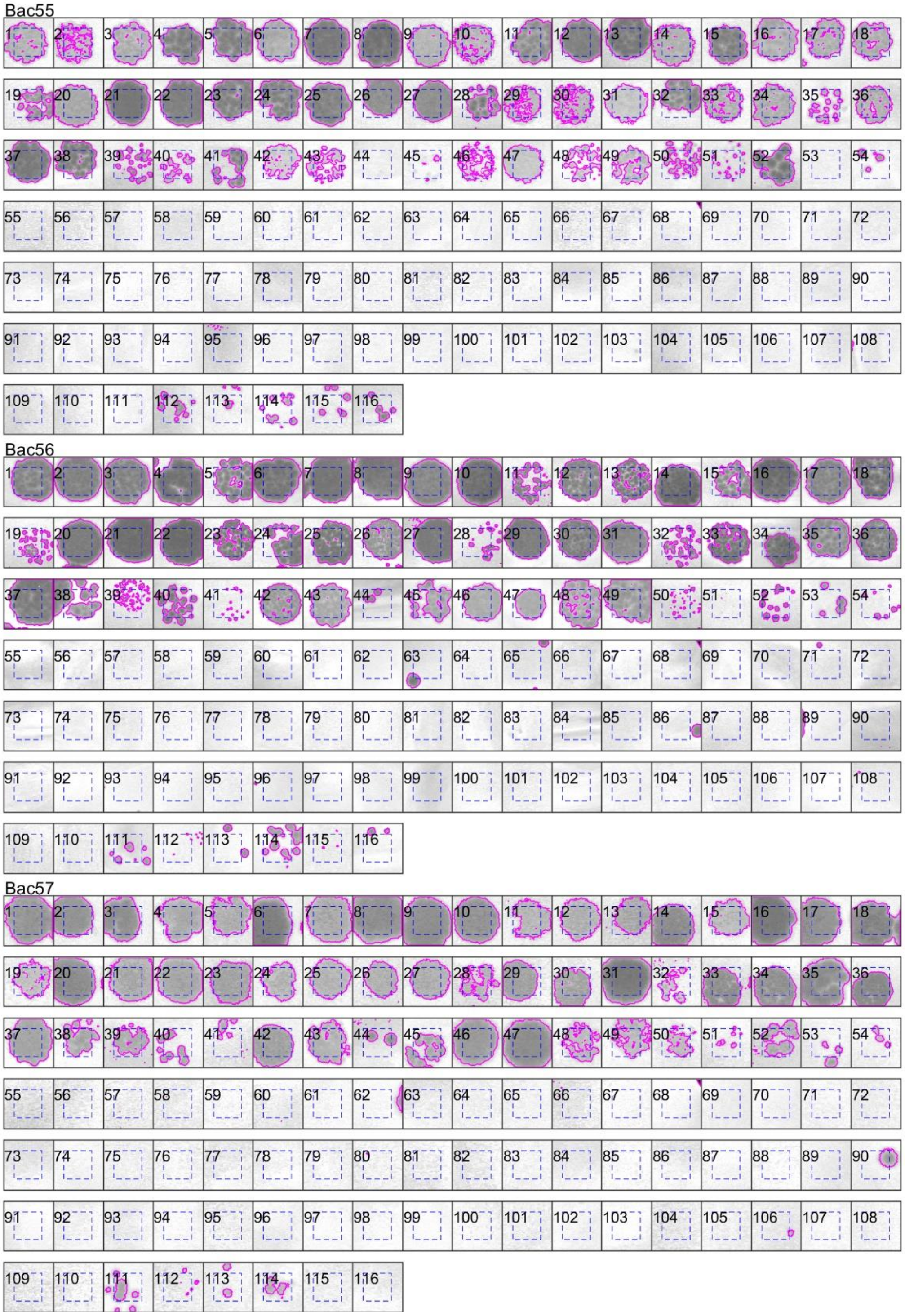

Supplementary Figure 11 - Bacteria-phage cross-infection interactions- raw data. **19/33**

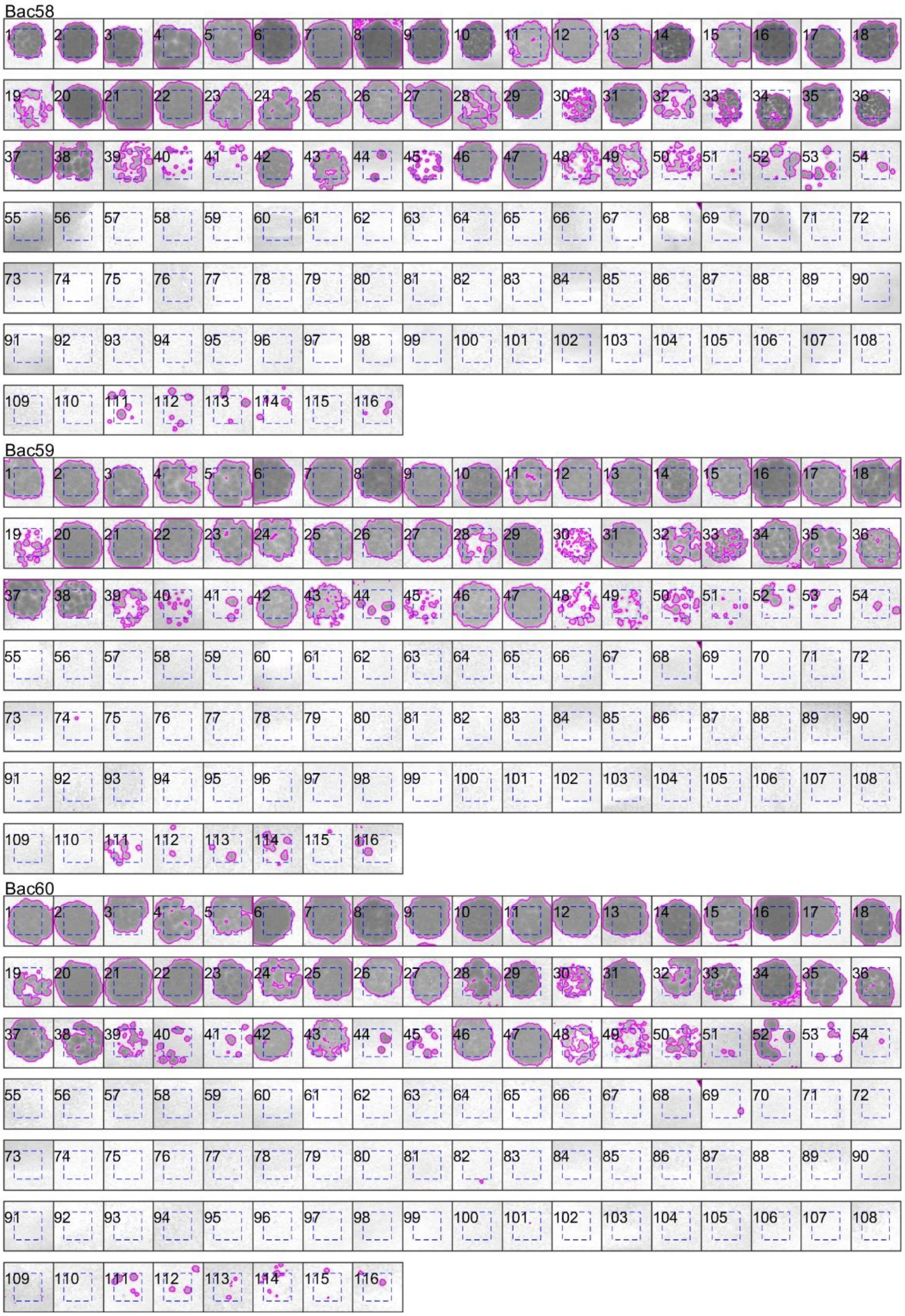

Supplementary Figure 11 - Bacteria-phage cross-infection interactions- raw data. **20/33**

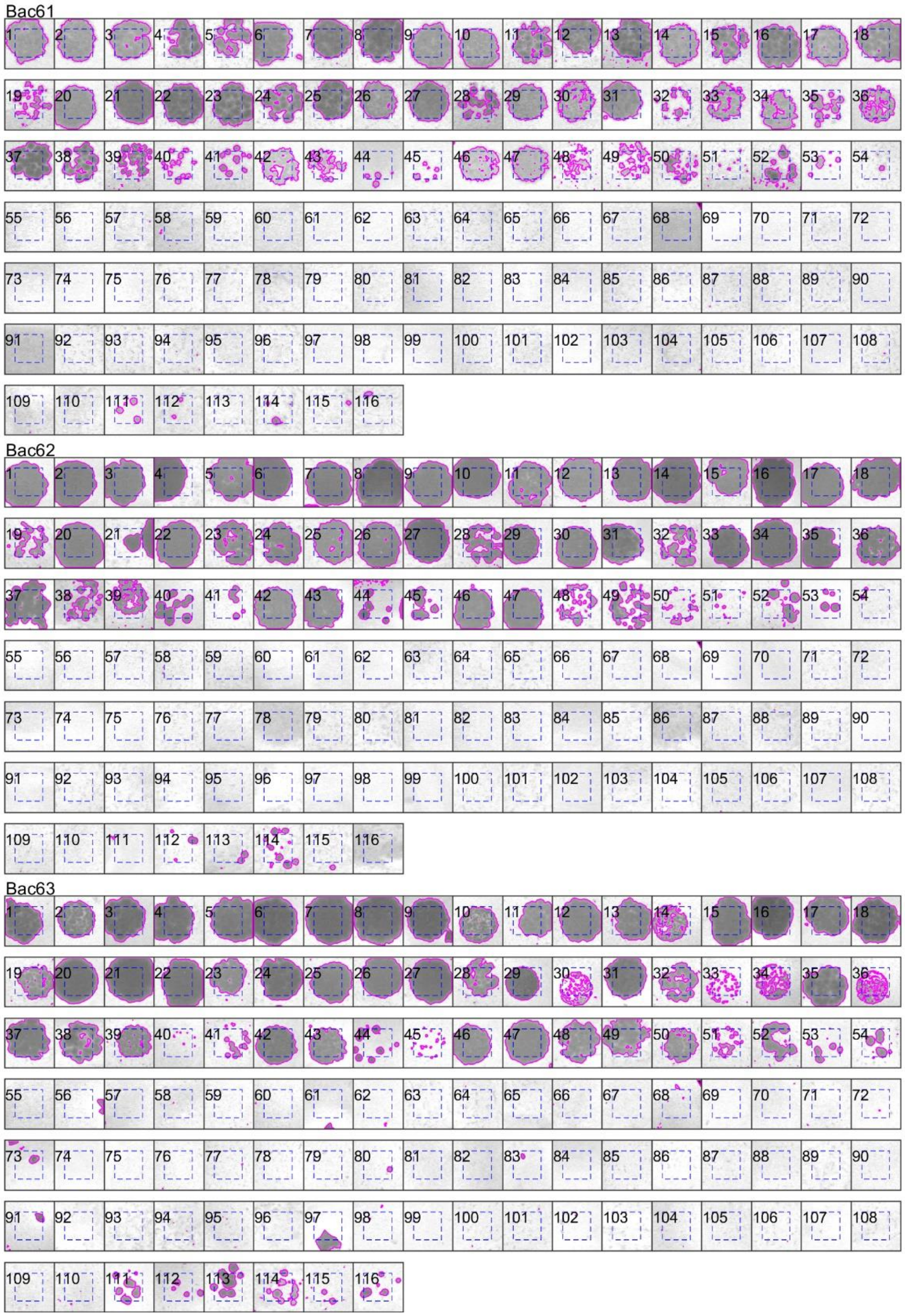

Supplementary Figure 11 - Bacteria-phage cross-infection interactions- raw data. **21/33**

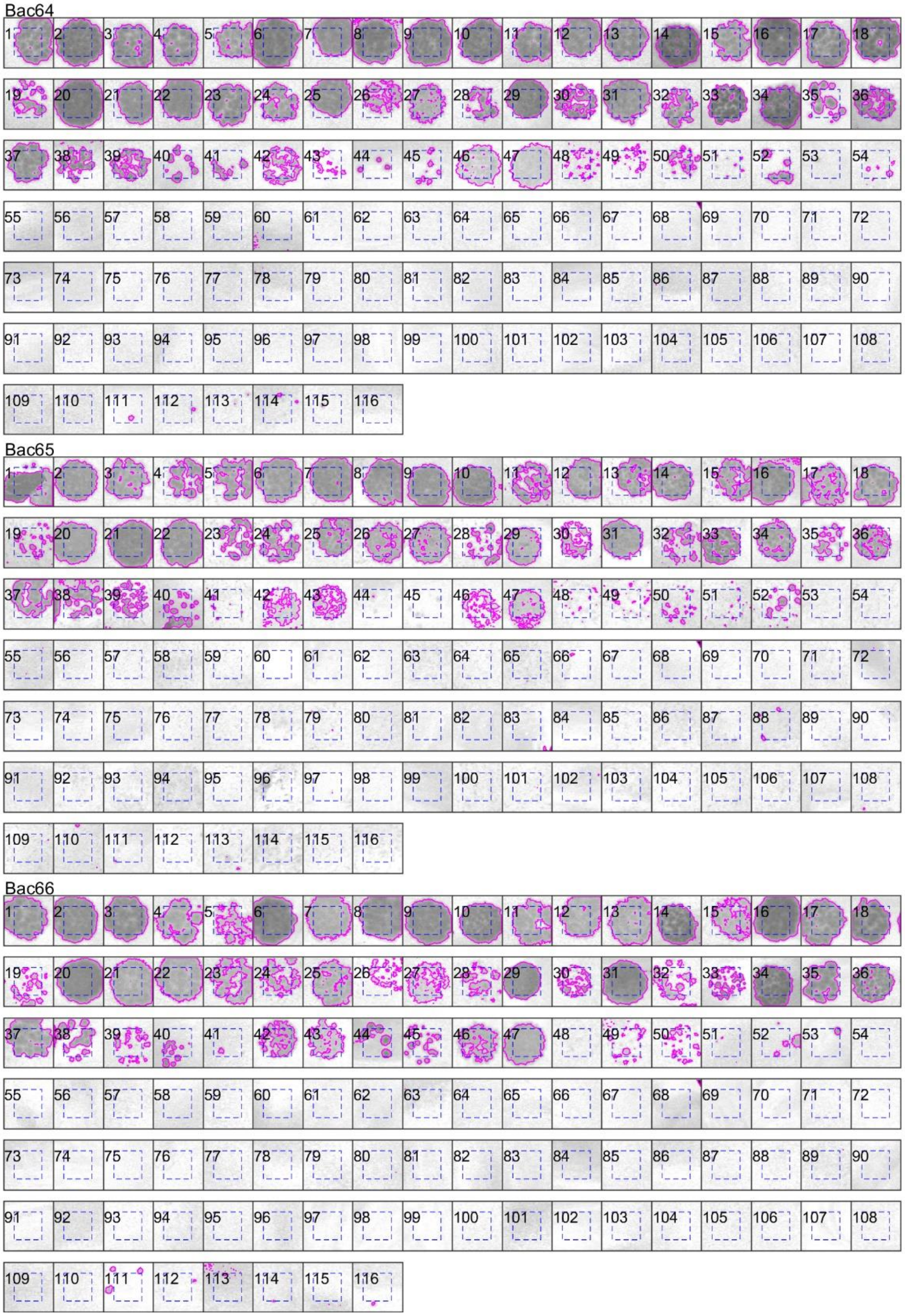

Supplementary Figure 11 - Bacteria-phage cross-infection interactions- raw data. **22/33**

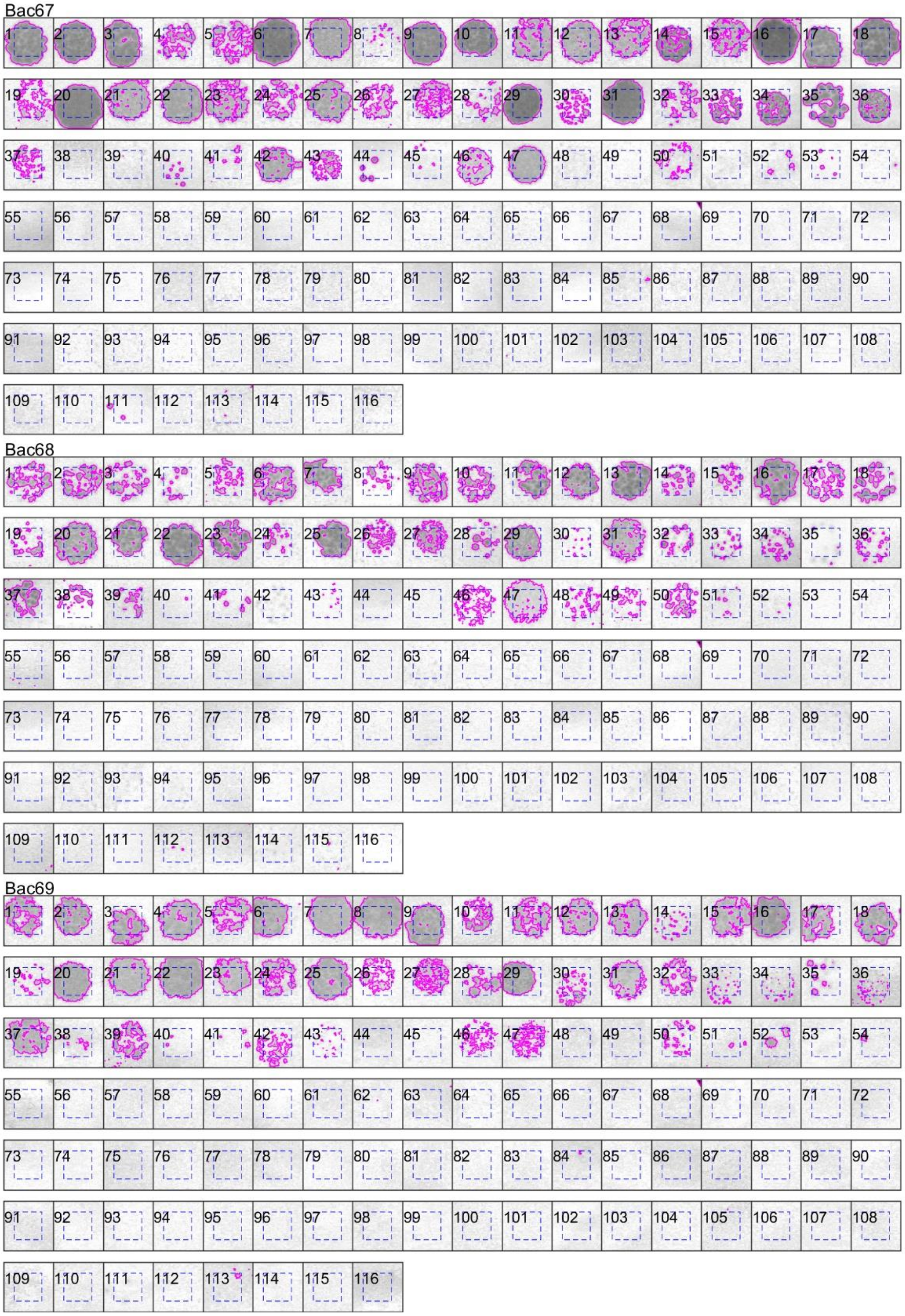

Supplementary Figure 11 - Bacteria-phage cross-infection interactions- raw data. **23/33**

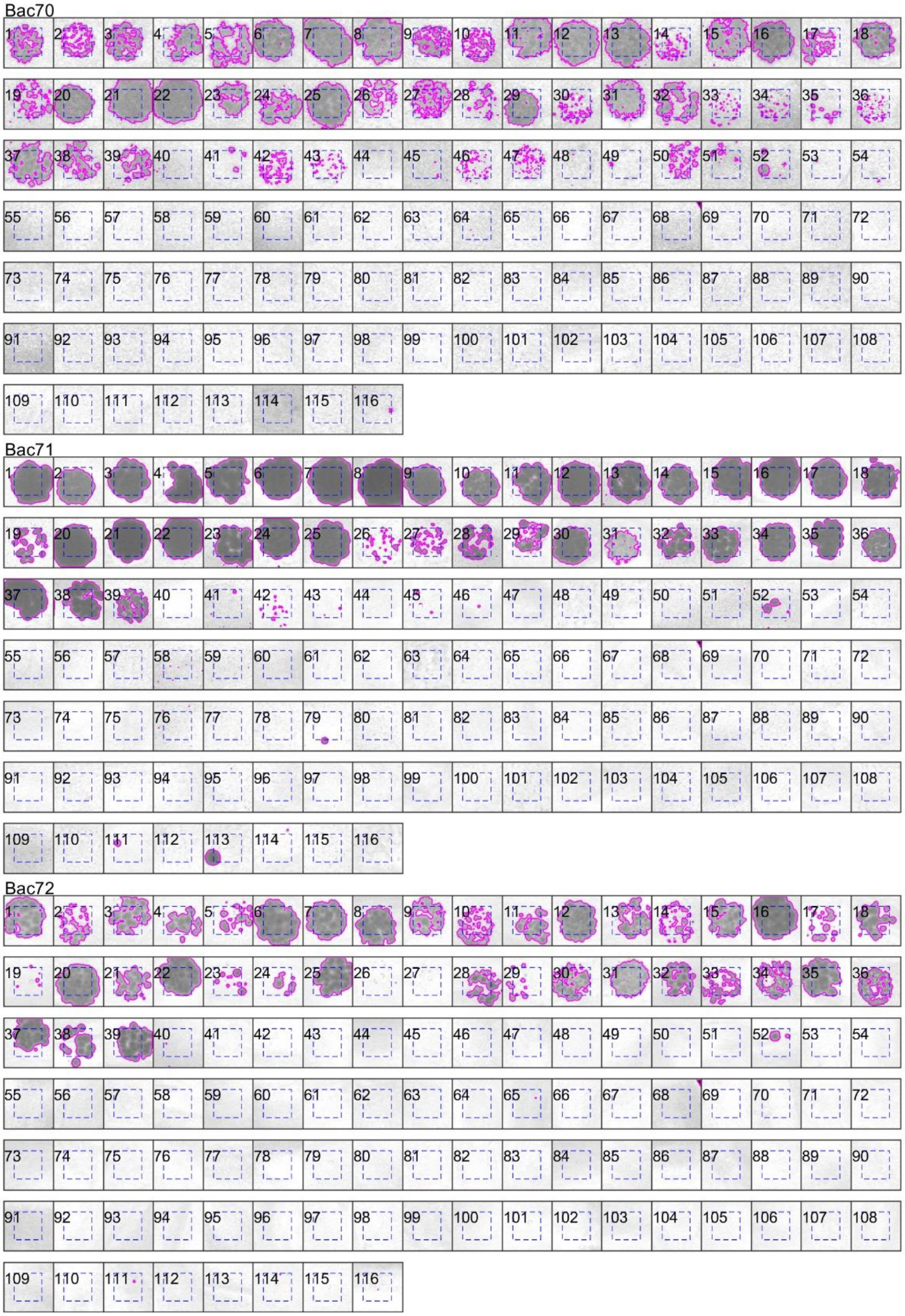

Supplementary Figure 11 - Bacteria-phage cross-infection interactions- raw data. **24/33**

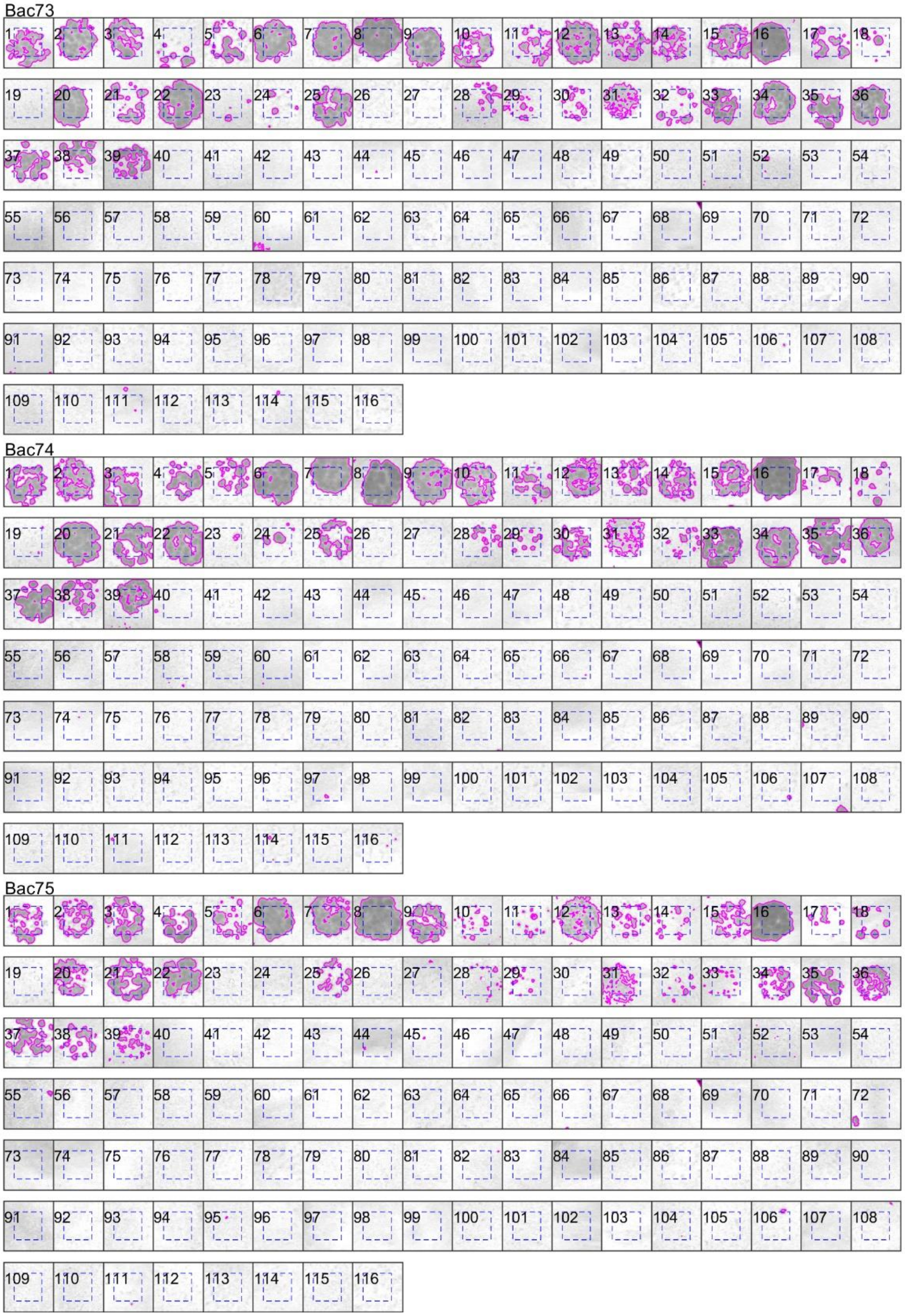

Supplementary Figure 11 - Bacteria-phage cross-infection interactions- raw data. **25/33**

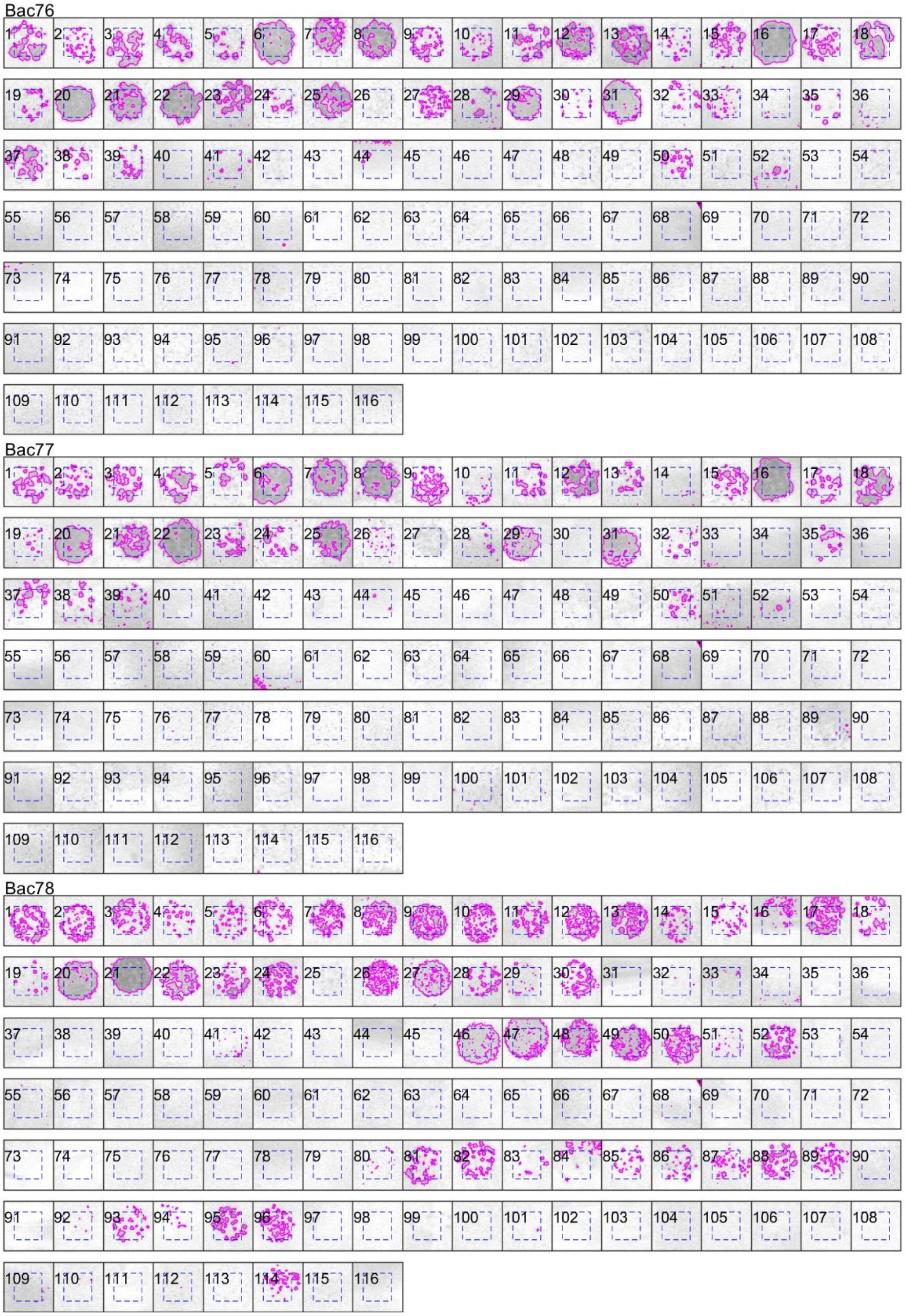

Supplementary Figure 11 - Bacteria-phage cross-infection interactions- raw data. **26/33**

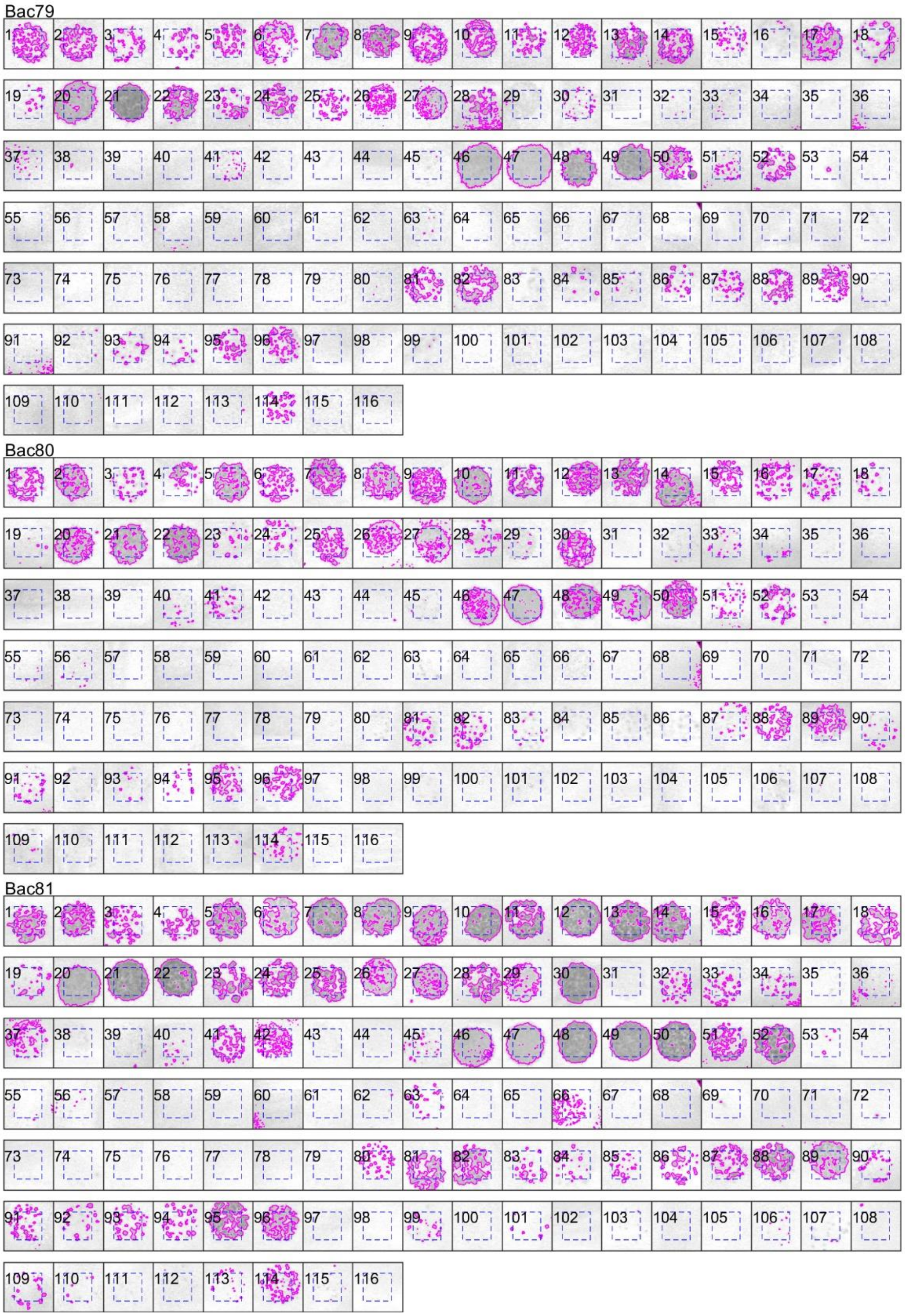

Supplementary Figure 11 - Bacteria-phage cross-infection interactions- raw data. **27/33**

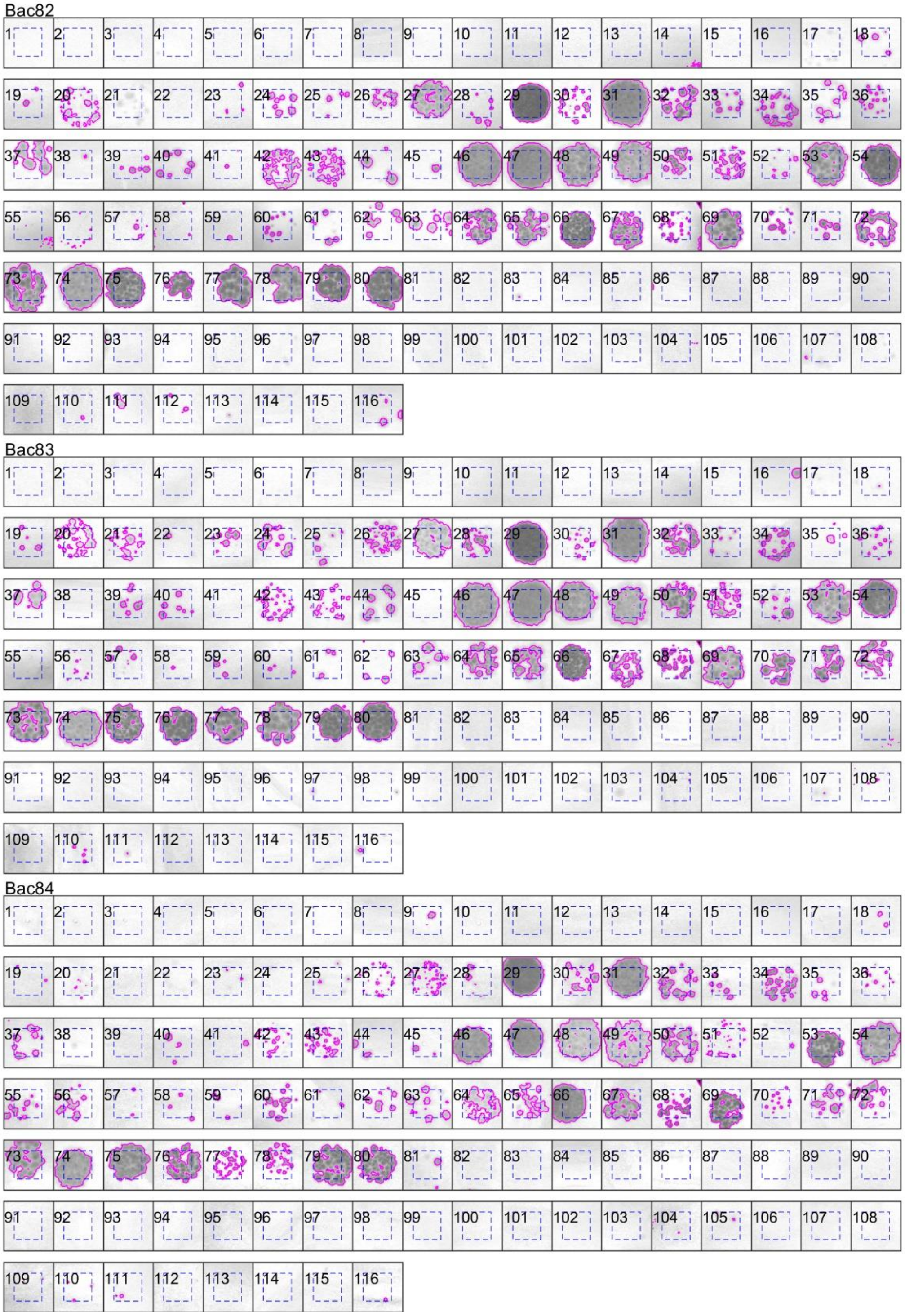

Supplementary Figure 11 - Bacteria-phage cross-infection interactions- raw data. **28/33**

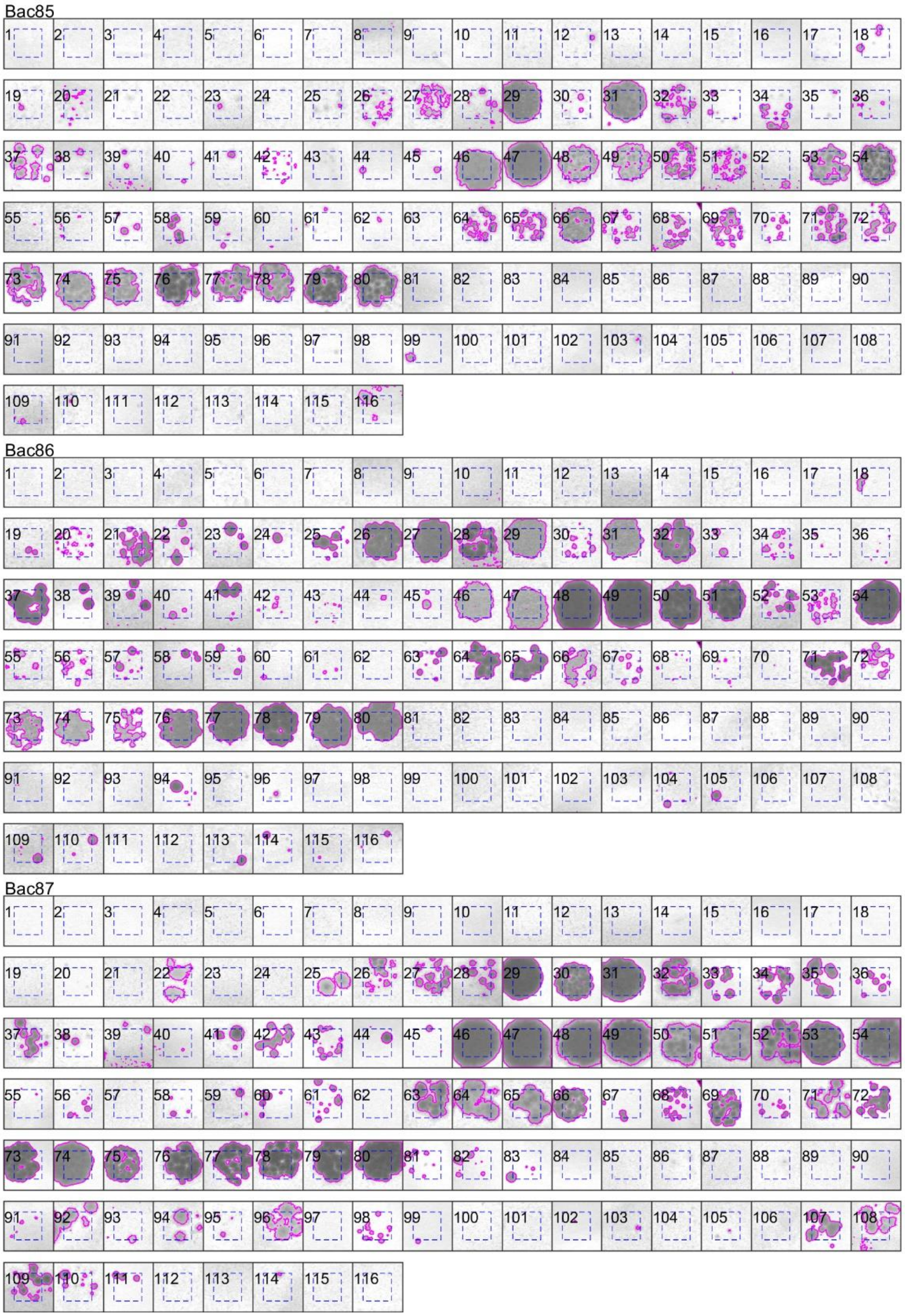

Supplementary Figure 11 - Bacteria-phage cross-infection interactions- raw data. **29/33**

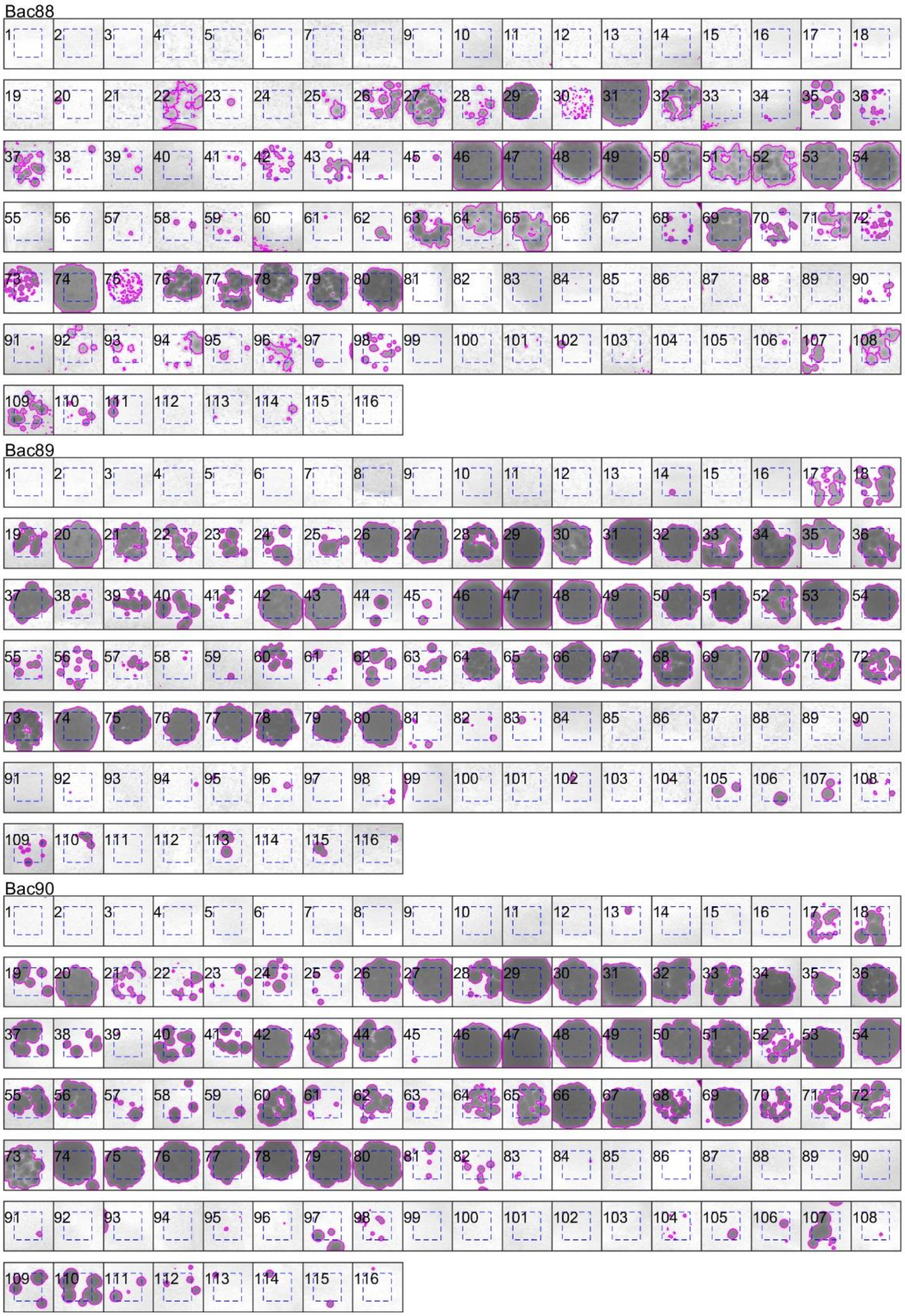

Supplementary Figure 11 - Bacteria-phage cross-infection interactions- raw data. **30/33**

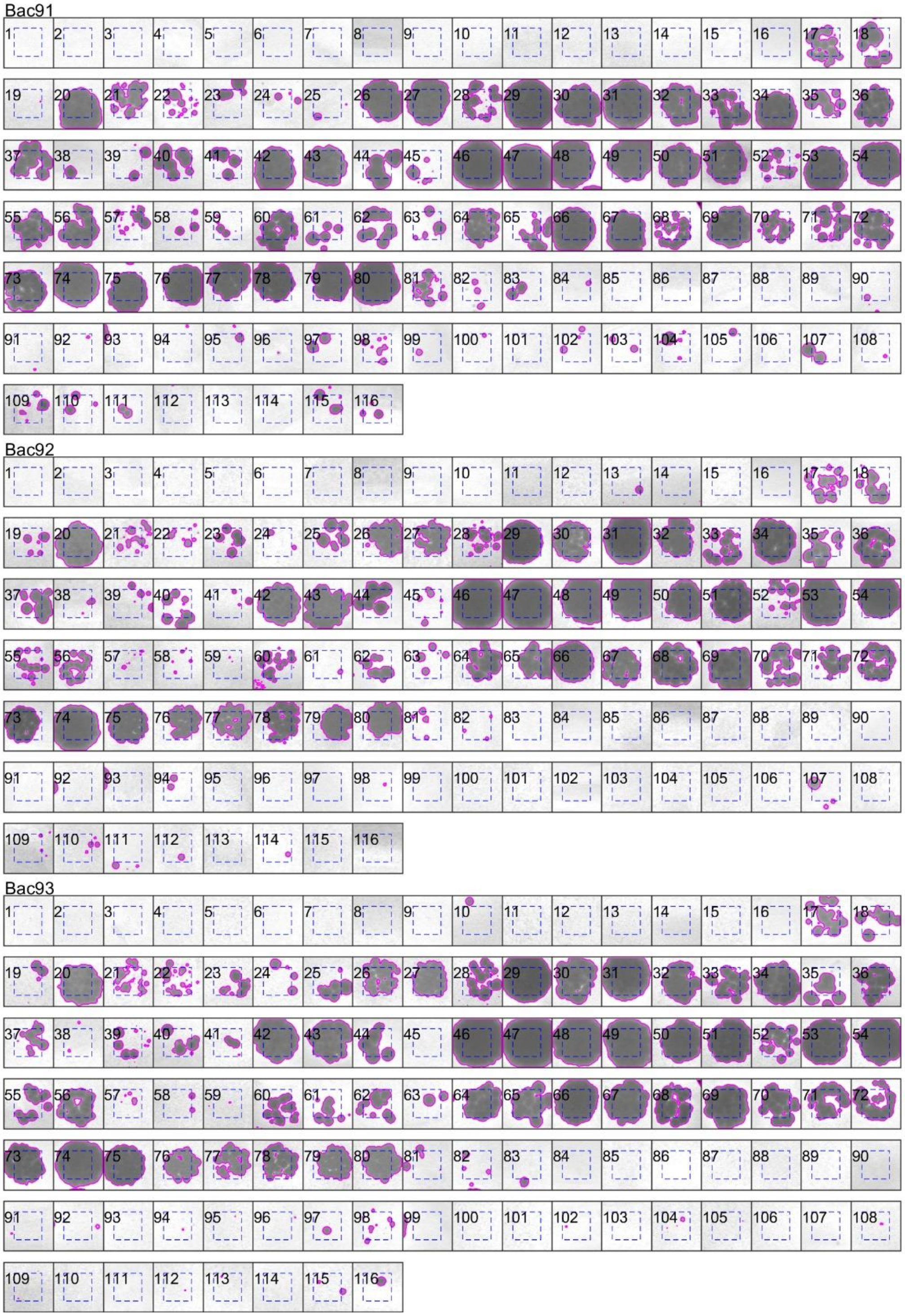

Supplementary Figure 11 - Bacteria-phage cross-infection interactions- raw data.

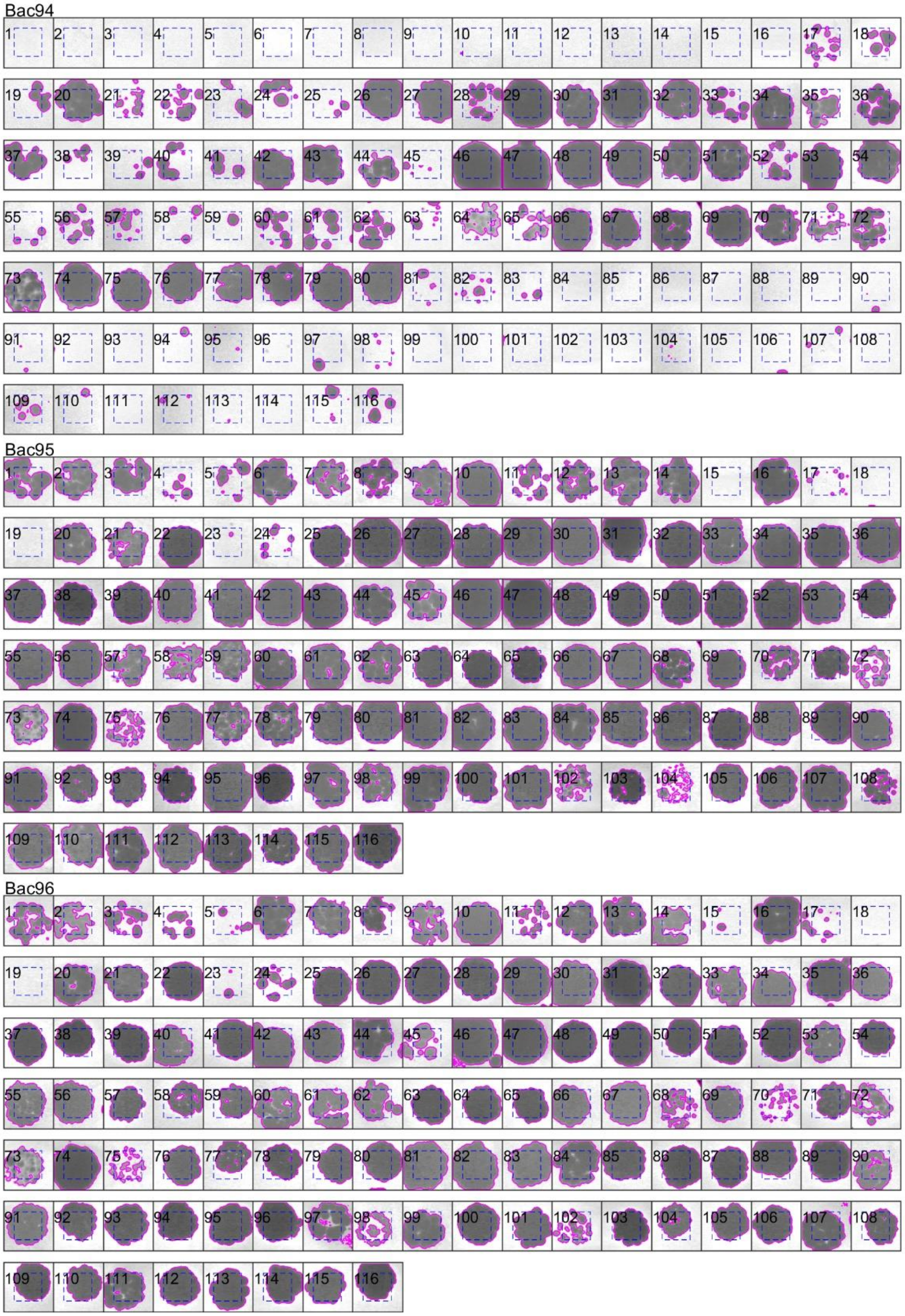

Supplementary Figure 11 - Bacteria-phage cross-infection interactions- raw data. **32/33**

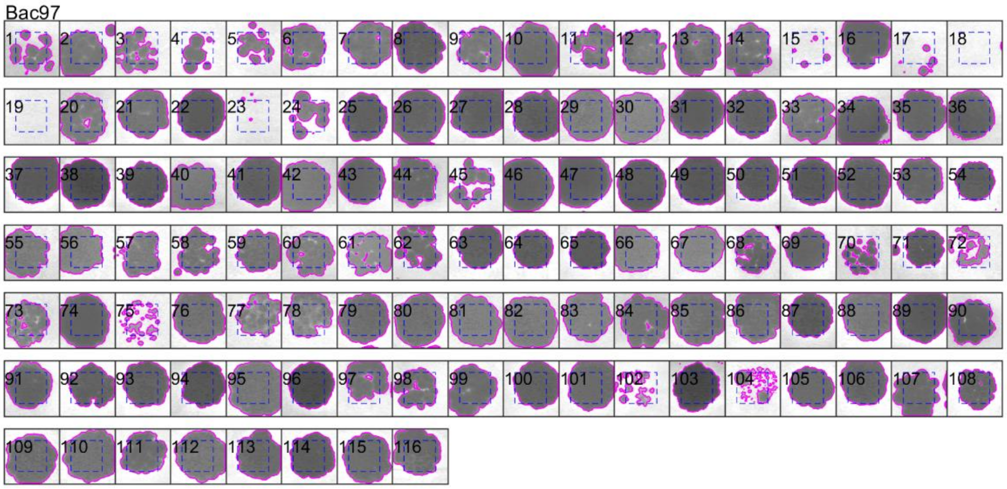

Supplementary Figure 11 - Bacteria-phage cross-infection interactions- raw data. **33/33**

References

1. Flores, C. O., Poisot, T., Valverde, S. & Weitz, J. S. BiMat: a MATLAB package to facilitate the analysis of bipartite networks. *Methods Ecol. Evol.* **7**, 127–132 (2016).
2. Falchi, F. A. *et al.* Mutation and Suppressor Analysis of the Essential Lipopolysaccharide Transport Protein LptA Reveals Strategies To Overcome Severe Outer Membrane Permeability Defects in Escherichia coli. *J. Bacteriol.* **200**, (2018).
