# Supplementary material for: Multistep diversification in spatiotemporal bacterial-phage coevolution
Source: Nat Commun. 2022 Dec 28;13:7971. doi: 10.1038/s41467-022-35351-w (PMC9797572; doi:10.1038/s41467-022-35351-w)
Supplement: Supplementary file 3 — Description of Additional Supplementary Files [file 41467_2022_35351_MOESM3_ESM.pdf]

Supplementary Data 1: Description of bacterial Isolates, with the following information: bacNms: Bacterial isolate number, chosen according to the order in the cross-infection matrix (Figure 2) and consistent across all tables and figures; SequenceNms: the name of the isolate sequencing fastq file; infectionIdx: the index of the isolate in the original cross-infection assay, for usage in the analysis pipeline; replicateNum: the plate replicate number from which this isolate was picked, from 1:4; experiment: whether this isolate was picked at the end of the initial (Init) or continual (Cont) coevolution round; coordinates_1,coordinates_2: coordinates in the original coevolution images from which this isolate was sampled, with (0,0) values for the wild type. isoNum: sample colony number (up to 2 colonies, A/B, were isolated from each sampled site).

Supplementary Data 2: Description of phage Isolates, with the following information: phgNms: Phage isolate number, chosen according to the order in the cross-infection matrix (Figure 2) and consistent across all tables and figures; SequenceNms: the name of the isolate sequencing fastq file; infectionIdx: the index of the isolate in the original cross-infection assay, for usage in the analysis pipeline; replicateNum: the plate replicate number from which this isolate was picked, from 1:4. Experiment:whether this isolate was picked at the end of the initial (Init) or continual (Cont) coevolution round; coordinates_1,coordinates_2: coordinates in the original coevolution images from which this isolate was sampled, with (0,0) values for the wild type. bacIsoNum: the number of the bacterial colony (A/B) on which this phage was isolated in the sample. isoNum: sample plaque number (up to 2 plaque , A/B, were isolated from each sampled site and bacterial colony); bacNms: the name of the bacterial isolate from which this phage plaque was isolated, as appears in Supplementary Table 1;

Supplementary Data 3: Bacterial mutation description, with the following information: mutName: short name of the mutation, as presented in Figure 3; Call: base change in case of a SNP; genomePos1: Start position of mutation in the reference genome (Genbank U00096.3); genomePos2: End position of mutation in the reference genome (Genbank U00096.3); InsSeq: in case of an insertion mutation not with a mobile element, this field will contain the inserted sequence; gene1/gene2: the index of the mutated gene in the reference genome (gene2 will contain a number if the mutation spreads across multiple genes); geneNm1/geneNm2: the name of the mutated gene (geneNm2 will contain a value if the mutation spreads across multiple genes); Length: mutation length in base pairs; ORFpos1: the position of the mutation within the open reading frame, will contain 0 in multi-gene mutations; AA: change in amino acid (in SNPs); mutType: mutation type; preexisting_loci: whether this mutation was detected in the wildtype strain (empty, relevant only to phages).

Supplementary Data 4: Phage mutation description, with the following information: mutName: short name of the mutation, as presented in Figure 3; Call: base change in case of a SNP; genomePos1: Start position of mutation in the reference genome (Genbank NC_001604); genomePos2: End position of mutation in the reference genome (Genbank NC_001604); InsSeq: in case of an insertion mutation not with a mobile element, this field will contain the inserted sequence; gene1/gene2: the index of the mutated gene in the reference genome (gene2 will contain a number if the mutation spreads across multiple genes); geneNm1/geneNm2: the name of the mutated gene (geneNm2 will contain a value if the mutation spreads across multiple genes); Length: mutation length in base pairs; ORFpos1: the position of the mutation within the open reading frame, will contain 0 in multi-gene mutations; AA: change in amino acid (in SNPs); mutType: mutation type; preexisting_loci: whether this mutation was detected in the wildtype strain.

Supplementary Data 5: Cross-infection: raw infection scores for Fig. 2. Each row is a bacterial isolate and each column is a phage isolate, ordered by hierarchical clustering as presented in Fig. 2a.

Supplementary Movie 1 - Time lapse movie of the initial coevolution experiment. Raw images were cropped around each plate's borders, the first image was subtracted from all subsequent images to remove background noise, and a set of 300 images with logarithmically increasing time gaps were assembled into a time lapse movie. The real time progression is displayed at the bottom time-bar.

Supplementary Movie 2 - Time lapse movie of the continual coevolution experiment. Raw images were cropped around each plate's borders, the first image was subtracted from all subsequent images to remove background noise, and a set of 340 images with logarithmically increasing time gaps were assembled into a time lapse movie. The real time progression is displayed at the bottom time-bar.
The observed non-symmetric propagation in the bottom-left replicate was a result of a drip during initial inoculation and not a contamination, as was later confirmed via sampling and genome sequencing.

Supplementary Movie 3 - No-phage control: Time lapse movie of bacterial growth and migration on swimming plates. Raw images were cropped around each plate's borders, the first image was subtracted from all subsequent images to remove background noise, and all images were assembled into a time lapse movie. Time progression is displayed at the bottom time-bar.
