# Supplementary material for: Multistep diversification in spatiotemporal bacterial-phage coevolution
Source: Nat Commun. 2022 Dec 28;13:7971. doi: 10.1038/s41467-022-35351-w (PMC9797572; doi:10.1038/s41467-022-35351-w)
Supplement: Supplementary file 12 — Reporting Summary [file 41467_2022_35351_MOESM12_ESM.pdf]

nature portfolio

Corresponding author(s): Roy Kishony

Last updated by author(s): Nov 23, 2022

Reporting Summary

Nature Portfolio wishes to improve the reproducibility of the work that we publish. This form provides structure for consistency and transparency in reporting. For further information on Nature Portfolio policies, see our Editorial Policies and the Editorial Policy Checklist.

Statistics

For all statistical analyses, confirm that the following items are present in the figure legend, table legend, main text, or Methods section.

n/a	Confirmed	
☐	☒	The exact sample size (*n*) for each experimental group/condition, given as a discrete number and unit of measurement
☐	☒	A statement on whether measurements were taken from distinct samples or whether the same sample was measured repeatedly
☐	☒	The statistical test(s) used AND whether they are one- or two-sided<br>*Only common tests should be described solely by name; describe more complex techniques in the Methods section.*
☐	☒	A description of all covariates tested
☐	☒	A description of any assumptions or corrections, such as tests of normality and adjustment for multiple comparisons
☐	☒	A full description of the statistical parameters including central tendency (e.g. means) or other basic estimates (e.g. regression coefficient) AND variation (e.g. standard deviation) or associated estimates of uncertainty (e.g. confidence intervals)
☐	☒	For null hypothesis testing, the test statistic (e.g. *F*, *t*, *r*) with confidence intervals, effect sizes, degrees of freedom and *P* value noted<br>*Give P values as exact values whenever suitable.*
☒	☐	For Bayesian analysis, information on the choice of priors and Markov chain Monte Carlo settings
☒	☐	For hierarchical and complex designs, identification of the appropriate level for tests and full reporting of outcomes
☒	☐	Estimates of effect sizes (e.g. Cohen's *d*, Pearson's *r*), indicating how they were calculated

Our web collection on statistics for biologists contains articles on many of the points above.

Software and code

Policy information about availability of computer code

Data collection	Automatic imaging of the coevolution experiment was controlled with the EOS Utility Software (Canon).<br>Fluorescence imaging of agar plates of the cross-infection assay and the two-color plaque assay were acquired with a custom build Macroscope software.
Data analysis	Conversion of coevolution experiment images into movies was done using LRTimelapse 5.6.0 and Adobe Premiere Pro 2022.<br>DNA sequence reads were aligned with Bowtie 1.2.1.1, SNPs were detected with SAMtools 0.1.19 and BCFtools 0.1.19, insertions and deletions were identified using Breseq 0.32.0.<br>A custom MATLAB script (R2020a) was used to analyze the data, including analysis of the cross-infection assay images, comparative sequencing analysis, Lasso model for mutation mapping, all statistical tests and generation of figures and tables. This script uses the BiMat package to calculate modularity and nestedness of the infection matrix, and the ColorBrewer package for figure color schemes.

For manuscripts utilizing custom algorithms or software that are central to the research but not yet described in published literature, software must be made available to editors and reviewers. We strongly encourage code deposition in a community repository (e.g. GitHub). See the Nature Portfolio guidelines for submitting code & software for further information.

March 2021

Data

Policy information about availability of data

All manuscripts must include a data availability statement. This statement should provide the following information, where applicable:

- Accession codes, unique identifiers, or web links for publicly available datasets
- A description of any restrictions on data availability
- For clinical datasets or third party data, please ensure that the statement adheres to our policy

Genome sequencing data is deposited in the public SRA database, see accession code PRJNA884167 [https://www.ncbi.nlm.nih.gov/bioproject/PRJNA884167/] for phage sequencing data and accession code PRJNA884682 [https://www.ncbi.nlm.nih.gov/bioproject/?term=PRJNA884682] for bacterial sequencing data. All other datasets generated during and analyzed during the current study were deposited in the Zenodo repository [DOI 10.5281/zenodo.7347986]. Source data are provided with this paper.

Field-specific reporting

Please select the one below that is the best fit for your research. If you are not sure, read the appropriate sections before making your selection.

☒ Life sciences ☐ Behavioural & social sciences ☐ Ecological, evolutionary & environmental sciences

For a reference copy of the document with all sections, see nature.com/documents/nr-reporting-summary-flat.pdf

Life sciences study design

All studies must disclose on these points even when the disclosure is negative.

Sample size	In order to sample diverse phenotypes, we sampled a total of 52 spots from the end point of all replicates of the initial and continual evolution rounds. This sample size was chosen as it included multiple distinct colony shapes and areas with various dynamic behavior on all plate replicates. From these samples we were able to isolate 104 bacterial isolates (2 colonies per sample and) and 188 phage isolates (up to 2 phages per bacterial isolate). We chose to isolate more than one colony and one phage per sample in order to identify local phenotypic and genotypic variability.
Data exclusions	Bacterial isolates were excluded from the analysis if they were expressing CFP or if they had low sequencing quality. Phage isolates were excluded if they were isolated from a CFP expressing bacterial isolate, if they had low sequencing quality, if they were suspected for cross-contamination during isolation or sequencing or if merging of two adjacent phage isolate drops occurred on the plate during the cross-infection assay.
Replication	Coevolution was performed in 4 replicates that showed qualitatively similar spatiotemporal dynamics. A preliminary coevolution experiment with similar strains and conditions on a single larger plate (30cmX30cm) showed similar results in terms of spatiotemporal dynamics, genetic diversity, and host switch evolution.
Randomization	Randomization was not relevant to our study, the infection of all phage isolates was tested against all bacterial isolates.
Blinding	Blinding was not relevant to our study, the infection of all phage isolates was tested against all bacterial isolates under the same conditions.

Reporting for specific materials, systems and methods

We require information from authors about some types of materials, experimental systems and methods used in many studies. Here, indicate whether each material, system or method listed is relevant to your study. If you are not sure if a list item applies to your research, read the appropriate section before selecting a response.

Materials & experimental systems

n/a	Involved in the study
☒	☐ Antibodies
☒	☐ Eukaryotic cell lines
☒	☐ Palaeontology and archaeology
☐	☒ Animals and other organisms
☒	☐ Human research participants
☒	☐ Clinical data
☒	☐ Dual use research of concern

Methods

n/a	Involved in the study
☒	☐ ChIP-seq
☒	☐ Flow cytometry
☒	☐ MRI-based neuroimaging

Animals and other organisms

Policy information about studies involving animals; ARRIVE guidelines recommended for reporting animal research

Laboratory animals	Escherichia coli K-12 strain MG1655 and bacteriophage T7.
Wild animals	*Provide details on animals observed in or captured in the field; report species, sex and age where possible. Describe how animals were caught and transported and what happened to captive animals after the study (if killed, explain why and describe method; if released, say where and when) OR state that the study did not involve wild animals.*
Field-collected samples	*For laboratory work with field-collected samples, describe all relevant parameters such as housing, maintenance, temperature, photoperiod and end-of-experiment protocol OR state that the study did not involve samples collected from the field.*
Ethics oversight	*Identify the organization(s) that approved or provided guidance on the study protocol, OR state that no ethical approval or guidance was required and explain why not.*

Note that full information on the approval of the study protocol must also be provided in the manuscript.
